# Polymorphic potential of SRF binding site of *c-Fos* gene promoter: *in vitro* study†

**DOI:** 10.1039/d4ra05897f

**Published:** 2024-12-03

**Authors:** Barbora Profantová, Václav Římal, Václav Profant, Ondřej Socha, Ivan Barvík, Helena Štěpánková, Josef Štěpánek

**Affiliations:** a Institute of Physics, Faculty of Mathematics and Physics, Charles University Ke Karlovu 5, 121 16 Prague 2 Czech Republic profant@karlov.mff.cuni.cz +420 95155 1471; b Department of Low-Temperature Physics, Faculty of Mathematics and Physics, Charles University V Holešovičkách 2, 180 00 Prague 8 Czech Republic

## Abstract

Recently published *in vivo* observations have highlighted the presence of cruciform structures within the genome, suggesting their potential significance in the rapid recognition of the target sequence for transcription factor binding. In this *in vitro* study, we investigate the organization and stability of the *sense* (coding) strand within the Serum Response Element of the *c-Fos* gene promoter (*c-Fos* SRE), specifically focusing on segments spanning 12 to 36 nucleotides, centered around the CArG-box. Through a thorough examination of UV absorption patterns with varying temperatures, we identified the emergence of a remarkably stable structure, which we conclusively characterized as a hairpin using complementary ^1^H NMR experiments. Our research decisively ruled out the formation of homoduplexes, as confirmed by supplementary fluorescence experiments. Utilizing molecular dynamics simulations with atomic distance constraints derived from NMR data, we explored the structural intricacies of the compact hairpin. Notably, the loop consisting of the six-membered A/T sequence demonstrated substantial stabilization through extensive stacking, non-canonical inter-base hydrogen bonding, and hydrophobic clustering of thymine methyl groups. These findings suggest the potential of the *c-Fos* SRE to adopt a cruciform structure (consisting of two opposing hairpins), potentially providing a topological recognition site for the SRF transcription factor under cellular conditions. Our results should inspire further biochemical and *in vivo* studies to explore the functional implications of these non-canonical DNA structures.

## Abstract 2

We show that the single-strand DNA of *c-Fos* promoter forms a highly stable hairpin. Its six-membered loop is stabilized through stacking, hydrogen bonding, and hydrophobic packing. This structure could facilitate recognition by transcription factors.

## Graphical abstract

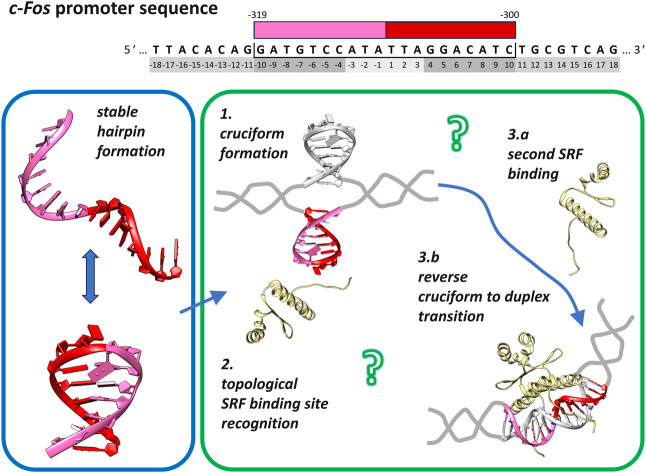

## Introduction

Although the concept of DNA cruciform formation was proposed long ago,^1^ it was not until the 1980s that cruciforms gathered significant attention, following the experimental validation through *in vitro* investigation.^2–4^ A crucial prerequisite for the successful extrusion of a cruciform involves the presence of two inverted repeat sequences (IRs) within the DNA double-helix sequence, which constitute the stems of the side arms in the cruciform structure. These arms terminate with loops composed of individual single strands situated between the IRs. The length of IRs should typically comprise at least six or seven base pairs.^5–8^ Additionally, the presence of A/T-rich regions within the gap between the IRs enhances the cruciform formation probability.^5^

Comparatively, the cruciform structure is considered energetically less favorable than a linear DNA double-helix due to the occurrence of unpaired and unstacked bases in the loops, alongside distorted base-pairing at the four-way junction region.^9,10^ Nevertheless, the thermodynamic stability of the cruciform can be enhanced through the release of negative DNA supercoiling.^5,7^ Most cruciform observations have been conducted using short synthetic oligonucleotides with appropriate sequences incorporated into circular DNA plasmids, thus inducing DNA supercoiling.^7,9–15^ Recently, magneto-optical tweezers have also been shown to provide the necessary torque for cruciform formation.^16^

In contrast to the numerous *in vitro* observations of cruciforms, reports of endogenous cruciform formation^17–20^ are mostly limited to sequences containing IRs in plasmids. However, the extrusion of cruciforms in chromosomal DNA and their biological function remain poorly understood. Even though IRs conducive to cruciform formation are found in transcriptional regulatory regions, their precise function in transcription remains elusive.^21^ In any case, the movement of RNA polymerase contributes to negative supercoiling^22^ which seems to be favorable for the formation of cruciforms. Additionally, some other proteins have been identified to induce cruciform formation.^13^ Overall, these recent *in vivo* observations have shed light on the presence of cruciform structures within the genome, suggesting their potential significance in the rapid recognition of the target sequence for transcription factor binding.

**Fig. 1:**
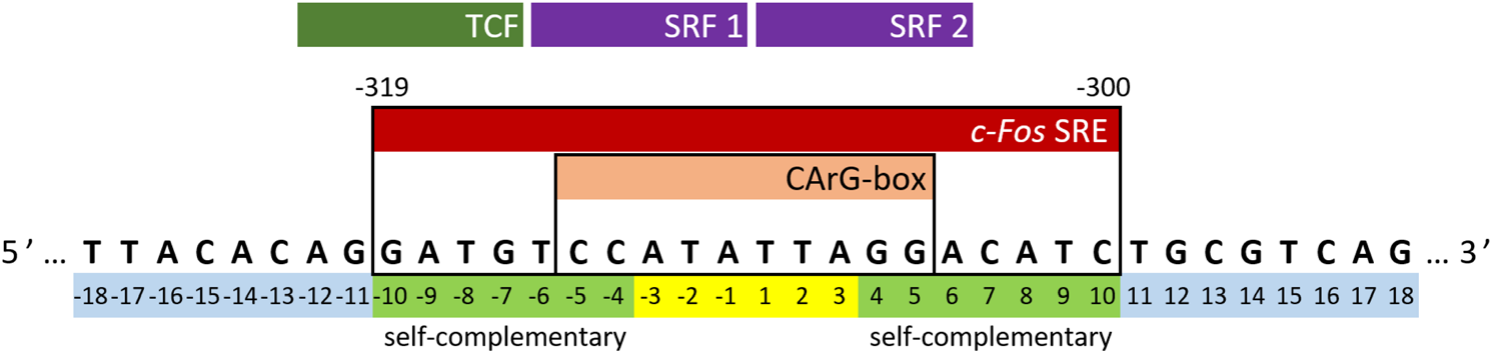
Sequence and our numbering of the *sense* strand of *c-Fos* SRE. The top row shows the positions of interacting proteins (two SRF molecules that form a dimer and a TCF). “Self-complementary” indicates parts that can form Watson–Crick pairs with each other.

The *c-Fos* proto-oncogene, categorized as an intermediate early gene (IEG), is a subject of extensive study due to its multiple regulatory elements crucial for orchestrating appropriate cellular responses.^23–25^ Transcriptional activation of the *c-Fos* gene involves several independent signaling cascades that target the upstream regulatory region of its promoter. A concise regulatory segment known as the serum response element (SRE) is of utmost significance among these cascades. It is both necessary and sufficient for the rapid induction of *c-Fos* gene expression.^26–29^ In the human *c-Fos* gene, the SRE spans from position −319 to −300, as depicted in Fig. 1. It encompasses a compact, imperfectly symmetrical element termed the CArG-box (CC-AT rich-GG-box), which mediates responsiveness to a multitude of stimulatory agents.^30,31^

The transcription factors that bind to the SRE include the ubiquitous serum response factor (SRF) and additional proteins referred to as ternary complex factors (TCF). These proteins form a ternary complex on the SRE in conjunction with SRF and DNA. SRF, which belongs to the MADS domain family of transcription factors,^32,33^ is a highly post-translationally modified 67 kDa protein that interacts with the CArG-box.^30,34^ SRF plays a role in the induction by various stimuli and is also implicated in the suppression of *c-Fos* gene expression.^35^ The known human TCF proteins^36^ (Elk-1, SAP-1, and NET) are members of the ETS transcription factors family and share a substantial degree of sequence homology.^37^ Notably, these factors do not bind independently to the *c-Fos* SRE but require the prior presence of prebound SRF to form the ternary complex.

Numerous studies have been conducted to characterize the interaction between SRF and SRE. The core domain of SRF (core-SRF) binds to the CArG-box as a homodimer, forming a specific SRF–SRE complex that induces an approximate 70° bend in the SRE double strand.^34,38,39^ Historically, the degree of SRE bending induced by protein binding has been regarded as the primary determinant for recognition by core-SRF.^34,40,41^ However, it has been demonstrated that SRE moieties in their free state exhibit continual interconversion between bent and linear conformations,^42,43^ a feature retained when complexed with core-SRF.^43^ Consequently, the recognition between the SRE and SRF was speculated to be more influenced by specific oscillations of the phosphate charge network stemming from SRE dynamics.^34,44^ However, all these studies were performed under conditions where the SRE segment was in duplex form. So far, no one has addressed the polymorphic potential of a single strand that is not associated with its complement.

The sequence of the *c-Fos* SRE possesses attributes compatible with a cruciform, comprising 7 base pairs (bp) of perfect IRs separated by a 6 bp region composed solely of A/T base pairs. In a putative cruciform structure, the six-membered A/T loops would be securely sealed by a tandem of G-C base pairs. Notably, the reversal of one of the G-C base pairs in the CArG-box has been observed to impede the binding of a second SRF molecule to the SRE.^34^ The notion of SRE adopting a cruciform structure as a target binding site for transcription factors has been previously speculated.^45^ In that study, the authors also demonstrated that an oligonucleotide with the SRE sequence undergoes partial cleavage at the A/T region when exposed to a single-strand-specific S1 nuclease. While this observation did not conclusively confirm the presence of a cruciform structure, it did indicate a temporary separation of strands in the A/T region.

Taken together, the research described above indicates a possible formation of cruciform in SRE. However, firm evidence is missing. To lay a solid ground for further discussions, we investigated the first necessary step required for cruciform formation: folding of one SRE strand into a stable hairpin. In our study, we conducted a comprehensive *in vitro* analysis of isolated SRE segments derived from the *sense* SRE strand. These sequences exhibited a remarkable ability to form highly stable hairpin structures, which could potentially act as the fundamental constituents of the *c-Fos* SRE cruciform. Our findings regarding hairpin formation were substantiated through a series of rigorous experiments, including UV absorption, fluorescence, and NMR spectroscopy. Furthermore, we meticulously explored the possibility of incomplete (mismatched) homoduplex formation. Our analysis revealed that the presence of such structures was minimal, constituting only a tiny fraction (a mere few percentage points) even under the millimolar concentrations used in our NMR experiments. Therefore, they did not significantly impact our findings and conclusions on hairpin formation. To further elucidate the stability and thermodynamic properties of the SRE hairpin, we performed a thorough investigation that involved melting analysis of both UV and NMR data. Additionally, we employed molecular dynamics simulations to propose a structural model for the SRE hairpin, leveraging inter- and intranucleotide distances obtained from NOESY NMR experiments.

## Material and methods

### Samples

For UV absorption experiments, the SRE oligodeoxynucleotides (ODN) – SREseg*N*, *N* = {16, 20, 28, 36} – were custom-synthetized by LMFR Masaryk University, Brno; the SREseg18 sample was obtained from the VBC-Biotech Company, Vienna, Austria. The samples were dissolved in phosphate buffer pH = 7 consisting of 20 mM Na_2_HPO_4_ and 20 mM NaH_2_PO_4_ with the addition of 100 mM NaCl. The total Na^+^ concentration was 160 mM. The concentrations of individual samples are given in Tables 1 and 2.

**Table 1:** Thermodynamic parameters of SREseg16 and reference octamers obtained from UV absorption and NMR temperature dependence measurements for the hairpin and duplex models

	Hairpin model		Duplex model			
	SREseg16 UV	SREseg16 NMR	SREseg16 UV	SREseg16 NMR	CTTCGAAG NMR	CTTGCAAG NMR
No. of nucleotides	16	16	16	16	8	8
No. of base pairs	5	5	12	12	8	8
*M*_w_ a (g mol^−1^)	4880	4880	4880	4880	2410	2410
*c* (μM)	3.08	1140	3.08	1140	1060	900
*T*_m_ (°C)	57.4 ± 0.4	60.8 ± 0.6	55.2 ± 0.2	59.6 ± 0.6	45.7 ± 0.6	47.8 ± 0.7
Δ*H* (kJ mol^−1^)	−137 ± 3	−137 ± 1	−217 ± 5	−211 ± 1	−252 ± 4	−236 ± 5
Δ*H*/bp (kJ mol^−1^)	−27	−27	−18	−18	−31	−29
Δ*S* (J mol^−1^ K^−1^)	−414 ± 10	−409 ± 3	−555 ± 14	−577 ± 4	−732 ± 12	−677 ± 16
Δ*S*/bp (J mol^−1^ K^−1^)	−83	−82	−46	−48	−92	−85

a Calculated using oligonucleotide properties calculator *Oligo Calc*^46^

**Table 2:** Thermodynamic parameters of SREseg*N* of various lengths obtained from UV absorption and NMR temperature dependence measurements using the hairpin model

	NMR			UV				
	SREseg12	SREseg14	SREseg16	SREseg16	SREseg18	SREseg20	SREseg28	SREseg36
*N*	12	14	16	16	18	20	28	36
No. of WC base pairs	3	4	5	5	6	7	7	7
*M*_w_ a (g mol^−1^)	3645	4263	4880	4880	5498	6116	8612	11 059
*c* (μM)	1000	1200	1140	3.08	4.44	4.07	2.68	2.13
*T*_m_ (°C)	32 ± 2	52.7 ± 0.6	60.8 ± 0.6	57.4 ± 0.4	60.5 ± 0.4	62.4 ± 0.5	68.0 ± 1.8	59.6 ± 0.7
Δ*H* (kJ mol^−1^)	−62 ± 2	−129 ± 1	−137 ± 1	−137 ± 3	−170 ± 4	−183 ± 5	−227 ± 7	−149 ± 7
Δ*H*/bp (kJ mol^−1^)	−21	−32	−27	−27	−28	−26	−32	−21
Δ*S* (J mol^−1^ K^−1^)	−204 ± 7	−397 ± 4	−409 ± 3	−414 ± 10	−508 ± 12	−546 ± 16	−664 ± 24	−448 ± 22
Δ*S*/bp (J mol^−1^ K^−1^)	−68	−99	−82	−83	−85	−78	−95	−64

a Calculated using oligonucleotide properties calculator *Oligo Calc*^46^

For NMR experiments, the SRE ODN – SREseg*N*, *N* = {12, 14, 16} – were purchased from ATDBio Ltd, Southampton, United Kingdom. The self-complementary DNA octamers used as reference duplexes (CTTGCAAG and CTTCGAAG) were obtained from Core Facility Proteomics of CEITEC – Central European Institute of Technology, Brno, Czech Republic. The samples were dissolved in 25 mM phosphate buffer in 90 : 10H_2_O : D_2_O, 200 mM Na^+^ in total, pH 7.0, reaching approximately 1 mM concentration of the ODN (Tables 1 and 2). Sodium 4,4-dimethyl-4-silapentane-1-sulfonate (DSS, Sigma–Aldrich) was used as an internal chemical-shift standard.

For fluorescence experiments, the ODNs (standard purification) purchased from Generi Biotech were labeled by Fluorescein (FAM) at 3′end and/or by Cyanine 3 (Cy3) at 5′end. Besides basic oligonucleotide SREseg16 and its ideal complement (cSREseg16), we also used doubly labeled self-complementary decamer (palindrome) 5′-CTGACGTCAG-3 as a control. The samples were dissolved in the same buffer as used in the UV measurements.

### Experimental methods

#### UV-vis absorbance spectroscopy

Absorption spectra of SREseg samples at concentrations shown in Table 2 were recorded on Lambda 12 (PerkinElmer, Inc.) UV-vis absorption spectrometer. The spectrometer was supplemented by an open-bath circulator Thermo Haake (Thermo Electron Corp.) which enabled precise temperature adjustment of measured samples within the studied 5–85 °C interval with approximately 4 °C step. Each spectrum was acquired in 220–320 nm wavelength region. The optical path length was 1 cm. The maximum absorbance was between 0.5 and 1 in all measurements.

#### NMR

The NMR experiments were carried out on a Bruker Avance III HD spectrometer with a proton frequency of 500.13 MHz. ^1^H spectra were acquired with water-signal suppression by excitation sculpting using pulsed-field gradients and selective 4 ms pulses.^47^ The total relaxation period was at least 2.3 s and the number of scans varied between 128 and 1024. The target temperature was set by using a flow of tempered dry nitrogen. Starting above 75 °C, the temperature was decreased, and the spectra were recorded for every 2 °C temperature drop. The acquisition started at least 15 min after reaching the desired temperature. Fourier transform with no window function was followed by zero- and first-order phase corrections and linear-baseline subtraction.

Resonances in the folded state were assigned from ^1^H–^1^H NOESY spectra, mixing time 200 ms (120 ms for SREseg12), with the water suppression described above. 512 rows, 128 scans (256 scans for SREseg12), each of 4096 data points were collected in States–TPPI mode at 13 °C for SREseg16, 15 °C for SREseg14, and 35 °C for SREseg12. Zero-filling, apodizations by squared cosines, Fourier transform, first-order phase correction, and 5-order baseline correction in both dimensions were performed by NMRPipe.^48^ The cross-peaks were assigned and integrated with Sparky.^49,50^ The inter-atomic distances were calculated from the integrals based on the sixth-power dependence^51^ and referenced to the average distance between CH5 and H6 as 2.9 Å.^52^

^1^H–^13^C HMBC experiments at natural abundance with WATERGATE^53^ were run to assign the adenine H2 signals by their connectivities to H8 through couplings with C4:^54^ besides a spectrum with 60 ppm wide carbon dimension fully covering the aromatic region, a ^1^H–^13^C HMBC with the carbon dimension narrowed down to 12 ppm and centered at 152 ppm, was employed. To improve the resolution in the indirect dimension and to avoid spectral folding at the same time, a selective (sinc-shaped 500 ms long at 100 mW power) ^13^C pulse was used to convert the single-quantum coherence into a double-quantum one. 800 scans were acquired in each of 128 rows in the States–TPPI mode.

Least-square fits of the resonance lines in the VT ^1^H spectra were conducted by Asymexfit,^55^ version 2.3, in MATLAB. SRE ODN spectra were fitted by Lorentzian lines (fast chemical exchange limit). The spectra of reference duplexes were fitted by line shapes assuming a general two-site chemical exchange. Asymexfit served to analyze the temperature profiles of chemical shifts as well. The chemical shift *δ*(*T*) at a given temperature is the population-weighted mean of the shifts of the folded and the unfolded state, which are assumed to follow linear temperature dependence as in eqn (S3) and (S4) in ESI.† Global thermodynamic values of the melting process were obtained from a joint least-squares fits of all aromatic chemical shifts (H2, H6, H8, M7).

#### Fluorescence measurements

The measurements were carried out using a fluorescence spectrometer Fluoromax 4 (Horiba) at ambient temperature (about 21 °C). The spectral width of slits was 2.5 nm, photomultiplier counting period was 0.2 s. Prior to the measurement, the solutions were subjected to annealing – heating up to 80 °C followed by slow cooling for 2 hours to an ambient temperature. Four spectra, optimized to individual fluorophores FAM and Cy3, were recorded for each sample: emission spectrum in the spectral region 499–681 nm excited at 495 nm (near the absorption maximum of FAM), excitation spectrum 403–515 nm detected at 520 nm (the emission maximum of FAM), emission spectrum 549–681 nm excited at 540 nm (near the absorption maximum of Cy3), and excitation spectrum 403–553 nm detected at 560 nm (the emission maximum of Cy3).

### Data treatment and analysis

#### Factor analysis – singular value decomposition

Generally, factor analysis (FA) methods enable distinguishing among individual spectral components in a property-dependent spectral series, *i.e.*, spectral alteration caused by thermally induced structural change in our case. The sets of experimental spectra were subjected to FA using the singular value decomposition (SVD) algorithm. Within SVD, a set of *n* spectra is decomposed into a set of orthogonal normalized spectral profiles *S*_*j*_ according to the following equation1
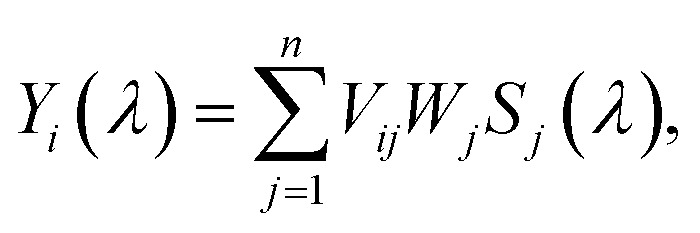
where the unitary matrix of normalized coefficients *V*_*ij*_ quotes relative portions of the *j*-th spectral profile *S*_*j*_ in the original spectra *Y*_*i*_. The diagonal matrix of singular values *W*_*j*_ stands for the statistical weights of spectral profiles. The spectral profiles are sorted by descending singular values until the terms at the right side of eqn (1) are sufficient to approximate the original spectral set within an experimental error. The number of necessary terms, *M*, the factor dimension, represents the number of independent spectral profiles resolved in the analyzed spectral set. Note that the first subspectrum *S*_1_ corresponds to the average, whereas other subspectra represent spectral changes (a detailed explanation of SVD can be found *e.g.*, in ref. 56).

#### Thermochemistry models

The structural transition observed both in UV absorption and NMR experiments may correspond to the melting of either hairpin or mismatch-duplex structure.

In the former case, the thermal transition between folded (closed hairpin, ss_h_) and unfolded (open hairpin, ss_un_) single-strand oligomer can be described as 
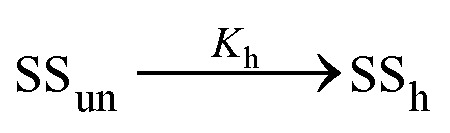
, with an association equilibrium constant *K*_h_ defined as2
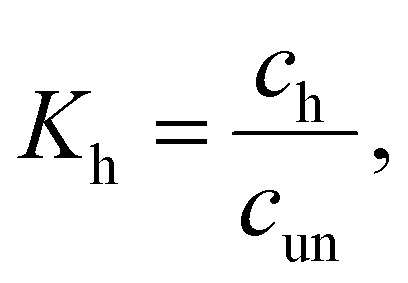
where *c*_un_ and *c*_h_ denote concentrations of the closed and open hairpin, respectively. *K*_h_ is defined thermodynamically in terms of change of Gibbs free energy Δ*G*_h_ (and subsequently also the changes of enthalpy Δ*H*_h_ and entropy Δ*S*_h_) as3
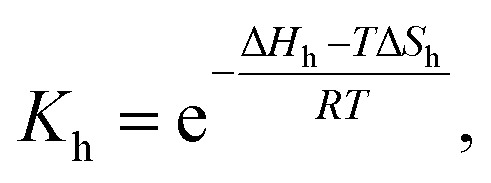
where *R* stands for molar gas constant and *T* is the thermodynamic temperature.

In the case of duplex formation, the thermal transition between folded (duplex) and unfolded (two single strands) form of oligomer can be described as 
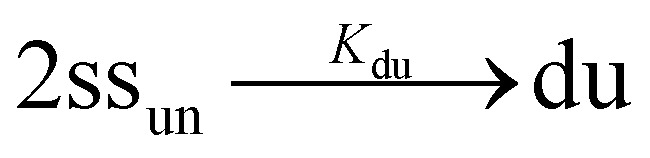
, with association equilibrium constant *K*_du_ defined as4
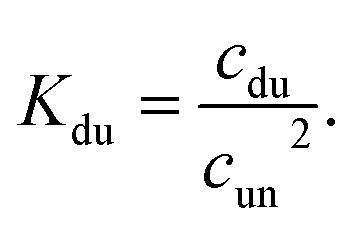
*K*_du_ is related to respective thermodynamic parameters by the formula5
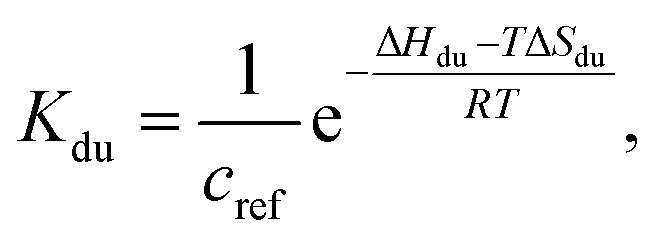
where *c*_ref_ is usually considered 1M.

We employed three models possessing relations for concentrations of folded and unfolded strands according to the total concentration of oligonucleotide strands *c*:

(i) Only hairpins are formed. Then6
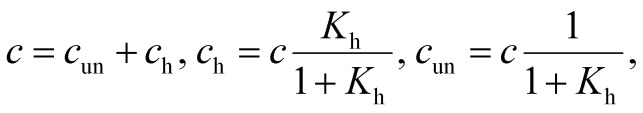
and the transition temperature is *T*_m_ = Δ*H*_h_/Δ*S*_h_.

(ii) Only duplexes are formed. Then7
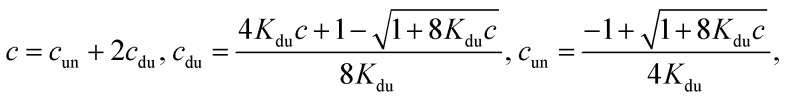
and 
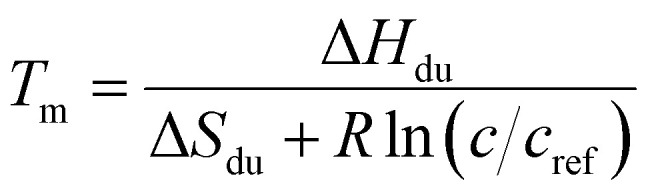
.

(iii) both hairpins and duplexes are formed. Then8
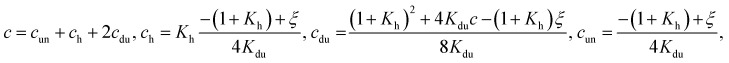
where 
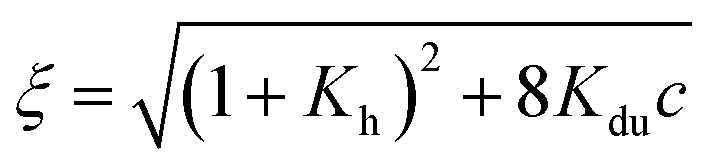
.

The explicit shape of the fitting equations for both UV absorption measurements and NMR is shown in the ESI† as well as the equations used for analysis of fluorescence experiments.

### Theoretical simulations

#### MD simulations

A tentative model of the SREseg16 hairpin was created through a short MD run with applied constraints corresponding to our NMR data. The initial structure was solvated using the TIP3P water model^57^ to ensure at least 10 Å of solvent in the periodic box and neutralized in 0.5 M NaCl (using VMD 1.9.3 (ref. 58)). This provided a periodic box with a size of ∼50 × 52 × 42 Å for a simulated system consisting of 9664 atoms. The CHARMM36 force field^59^ was used.^49,50^ MD simulations were produced with the aid of the software package NAMD 2.13.^60^ The simulated system was energy-minimized and heated to 300 K. Langevin dynamics was used for temperature control and the Langevin piston method was applied to reach an efficient pressure control with a target 1 atm.^60^ The integration time step was set to 2 fs. The SHAKE/SETTLE algorithm (tolerance 1 × 10^−8^) was applied to constrain bonds between each hydrogen and the atom to which it is bonded in nucleic acids and water molecules.^61^ The non-bonded cut-off was set to 12 Å. The MD run lasted for 100 ps. Data were recorded every 2 ps. The simulated system was visualized with the VMD 1.9.3 software package^58^ and analyzed using the CPPTRAJ module from the Amber Tools suite.^62^ The figures were produced using the UCSF Chimera software package.^63^

## Results and discussions

### Folding of SRE single strand into a hairpin

**Fig. 2:**
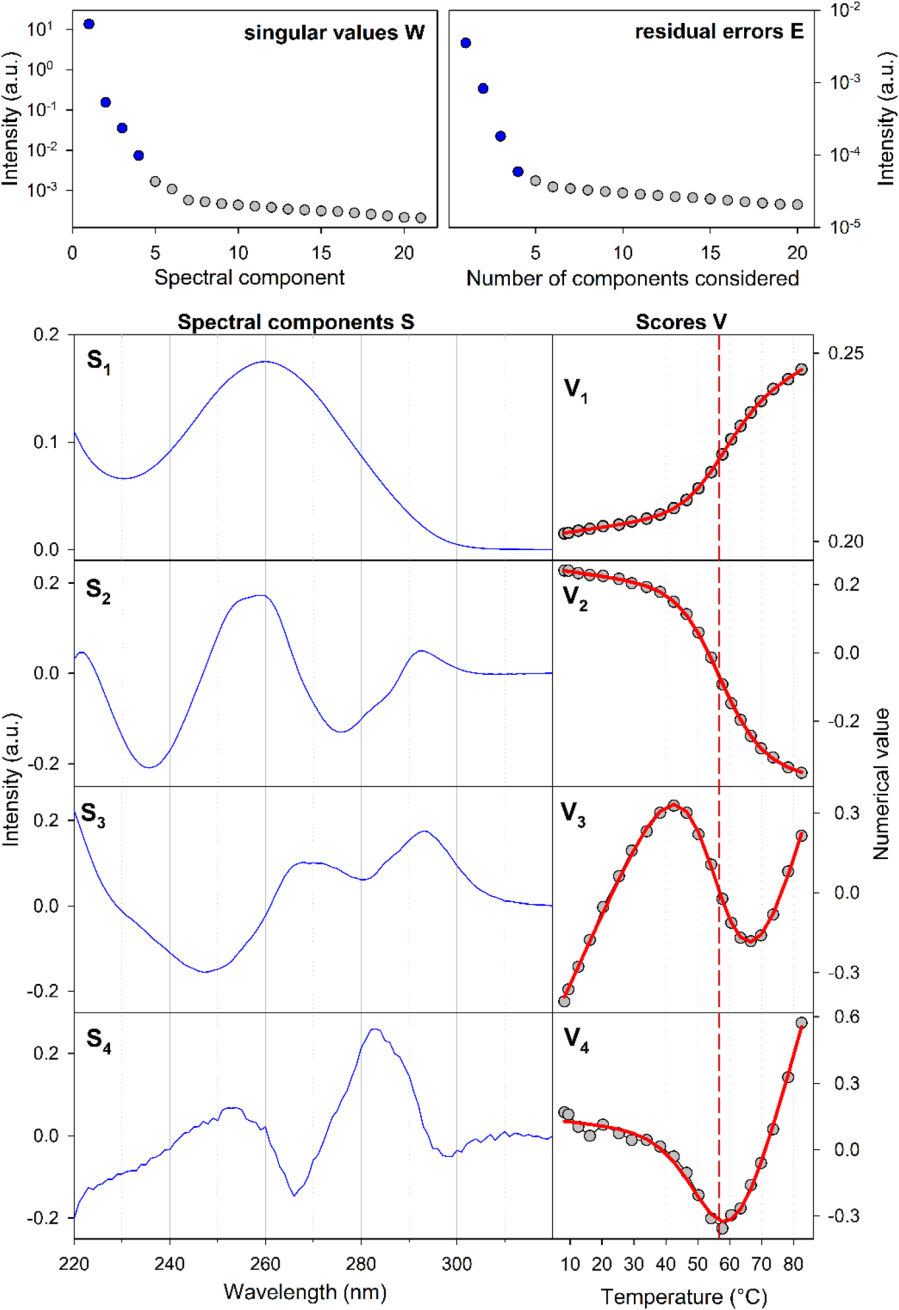
Temperature dependence of the SREseg16 UV absorption spectra: results of the singular value decomposition of the spectral series. The reaction equilibrium fit to the thermodynamic model of a single structural transition is shown in red (visually unresolvable curves were obtained for both considered cases, *i.e.*, hairpin and incomplete duplex). The dashed vertical line indicates the melting temperature obtained from the fit (see Table 1).

The experiments were conducted on segments of the *sense* SRE chain with varying lengths, denoted as SREseg*N*, where *N* represents the number of nucleotides; the nucleotide numbering is given in Fig. 1. Temperature-dependent UV absorption measurements were performed on segments ranging from 16 to 36 nucleotides at 2 to 4.5 μM concentrations. Notably, these experiments revealed a single distinct melting transition. In Fig. 2, we present the results of a singular value decomposition (SVD) analysis applied to a series of UV absorbance spectra collected over a temperature range of 5 to 85 °C for the SREseg16 segment, which spans from T-8 to A8 based on the nucleotide numbering (original spectra can be found in the ESI,† Fig. S1). The SVD data describes the dependence of the entire UV absorption spectra on temperature through a combination of spectral components and corresponding scores that represent their contributions to the spectra at different temperatures.

Our SVD analysis unambiguously demonstrates that a superposition of four distinct spectral components can accurately represent the spectral set. Several observations support this conclusion: (i) a substantial decrease in the singular values, signifying the statistical weight of individual spectral components, with the fifth singular value falling to less than one per mille of the first one, (ii) a decrease in the residual error as the number of considered spectral components increases, ultimately reaching a noise level with four components, and (iii) the erratic behavior of scores beyond the fifth spectral component (for the fifth and sixth components, refer to Fig. S2 in the ESI†). The presence of four spectral components aligns well with a straightforward temperature-induced transition between folded and unfolded forms. The first spectral component corresponds to the average spectral shape and its scores depict temperature-induced variations in overall absorbance, representing the standard melting curve. The remaining three components reveal relatively subtle changes in spectral shape. The second component dominates the transition-induced changes, exhibiting sigmoidal temperature dependence. In contrast, the third and fourth components predominantly capture linear changes below and above the transition temperature.

From the UV data alone, it is challenging to decisively distinguish whether the folded structure corresponds to a hairpin or an incomplete duplex; both scenarios could be adequately fitted to the melting data (see Table 1). Consequently, additional experiments were deemed necessary to clarify this aspect.

**Fig. 3:**
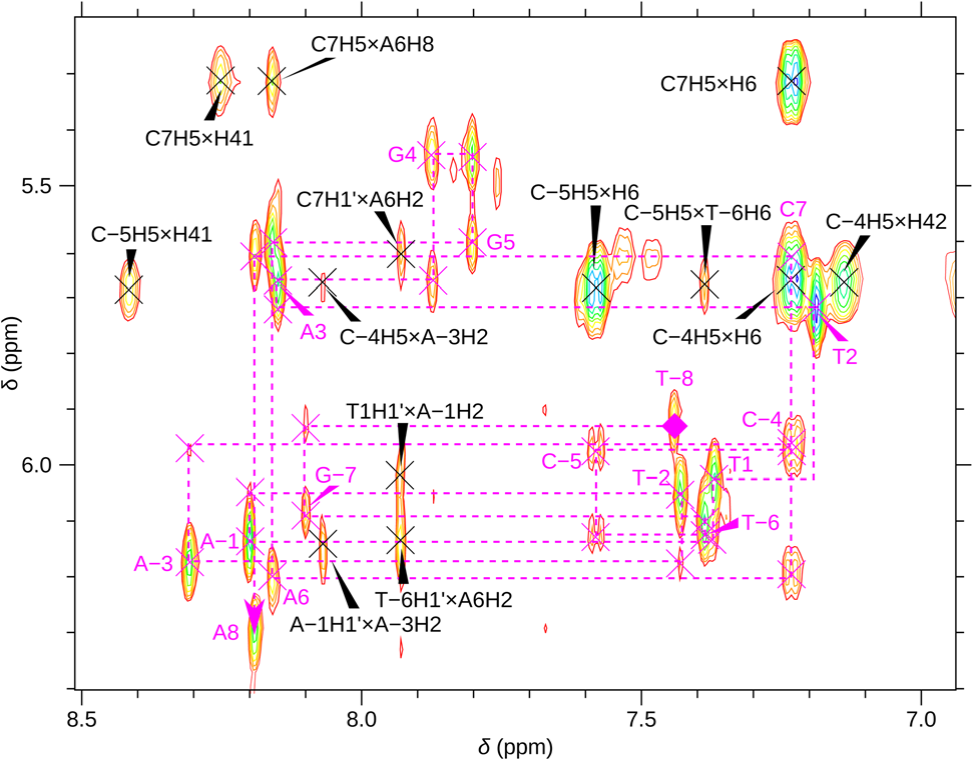
A part of the SREseg16 NOESY (13 °C). Sequential walk H1′–H6/H8 is depicted by the dashed magenta line, with the intranucleotide cross-peaks N*i*H1′ × N*i*H6/H8 labeled by the nucleotide identity. Selected cross-peaks not involved in this sequential walk are also shown.

**Fig. 4:**
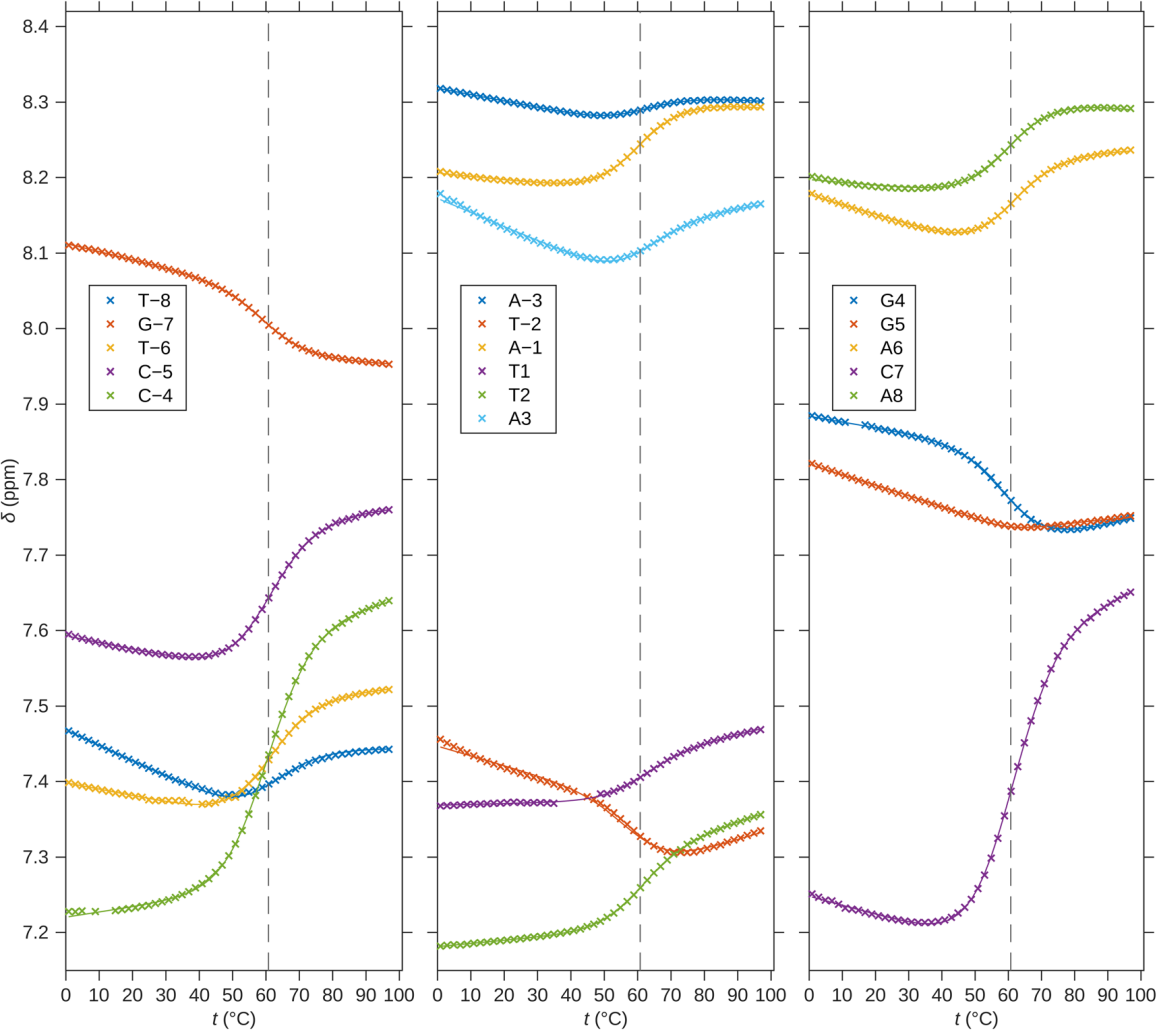
^1^H chemical shifts of H6/H8 nuclei of SREseg16. Solid lines correspond to fits of hairpin melting. Dashed vertical lines indicate the melting temperature obtained from the global fit.

For a better structural characterization, NMR spectroscopy was employed. We measured variable-temperature ^1^H NMR spectra of SREseg12, SREseg14, and SREseg16 and fully palindromic reference DNA octamers in ∼1 mM concentrations (Table 1). Individual ^1^H NMR lines were assigned by two-dimensional ^1^H–^1^H NOESY (Fig. 3) and ^1^H–^13^C HMBC (Fig. S3 in the ESI†). The obtained temperature dependences of aromatic proton chemical shifts were analogous to the UV results – a single distinctive sigmoid indicating a temperature-induced transition between folded and unfolded state was observed for each resonance (Fig. 4 and S4 in the ESI†). No significant differences between melting profiles from various parts of the oligodeoxynucleotide (ODN) are observed, indicating a two-state melting process. The melting temperature (60.8 °C, 1.14 mM ODN, Table 1) is very close to the value obtained from UV absorption (57.4 °C, 3.08 μM ODN), indicating a first-order folding, *i.e.*, hairpin formation.

The NMR results provide several clear arguments supporting the hypothesis that the dominant folded structure is a hairpin loop:

(i) The linewidths in the folded state of SREseg16 are very similar or in some cases even narrower in comparison to those observed for reference octamer duplexes (Fig. S5 in ESI†). As the linewidth is assumed to be given (under the same environmental conditions and when no chemical exchange takes place) by an inverse of the rotational correlation time that is proportional to the molecular size, it indicates that the folded structure of SREseg16 has the same molecular weight as the octamer duplexes. Therefore, it is very likely that the folded SREseg16 structure contains only one oligonucleotide strand.

(ii) No imino resonance from central thymines T-2, T1, and T2 has been detected. In a hypothetical duplex with such a relatively high *T*_m_, signals of these protons should be well visible even in mismatch pairs,^64,65^ including tandem mismatches.^66,67^ Therefore, a hairpin form that lacks stable hydrogen bonds involving the loop imino hydrogens^68^ is more likely.

(iii) The measured chemical shifts of aromatic protons in the central oligonucleotide region (N-4 through N4, including the last complementary pair CG_4) do not agree with the predictions for a mismatched duplex,^64,69,70^ while the predictions for the outer part of the ODN sequence suit our data well (Fig. S6 in ESI†).

(iv) The direction of temperature-induced changes of ^1^H chemical shifts in the central part of the folded state are often opposite to the reference double-helical octamers (Fig. S7 in ESI†).

(v) The folding enthalpy and entropy per Watson–Crick (WC) pair (Table 1) obtained from the hairpin model agree with the usual values (according to the reference duplexes in this study as well as in the literature), whereas these thermodynamic parameters based on the duplex model for SREseg16 (Table 1) reach only about 50–60% of the reference values. This observation comes from both UV and NMR data analysis.

In addition, the ^1^H NMR spectrum contains three unexpected exchangeable protons (6.694, 6.748, and 6.845 ppm at 11 °C) which we assign to adenine amino groups (AH61/62) involved in some non-canonical hydrogen bonds.

To conclude, the temperature dependencies of both UV absorption and ^1^H NMR show one folding transition with similar melting temperatures, *i.e.*, independent of the ODN concentration. The folded state was therefore indicated as a hairpin loop.

### Exclusion of a significant homoduplex presence in highly concentrated samples

**Fig. 5:**
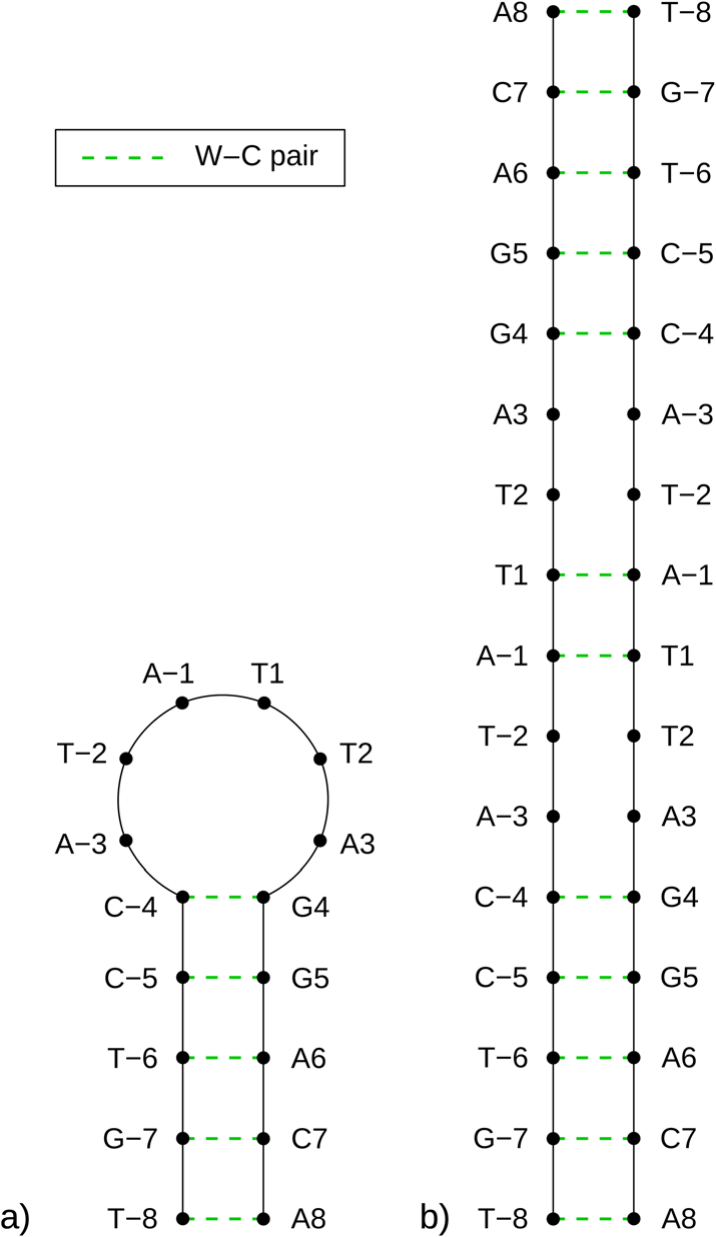
Schemes of possible base pairing in SREseg16 folded into a hairpin (a, left) or a mismatched duplex (b, right).

As previously mentioned, any partially palindromic sequence, including the SRE segments, has the potential to fold into one of two structural forms: a single-strand hairpin or a double-stranded incomplete (mismatched) homoduplex. For the SREseg16, the hairpin structure would consist of a stem comprising five WC base pairs (AT_8, CG_7, AT_6, CG_5, and CG_4) and a loop of six nucleobases (A-3, T-2, A-1, T1, T2, A3). Conversely, the duplex structure would be stabilized by twelve WC base pairs, with two consecutive pairs of mismatches occurring at A-3, T-2, and T2, A3 (see Fig. 5).

Despite identifying the hairpin as the prevailing structure, the folding/unfolding of which is expressed by the sigmoidal deviation of temperature dependencies, the simultaneous presence of homoduplexes has yet to be completely excluded. Since homoduplexes represent bimolecular complexes, their presence is expected to be more prominent in highly concentrated solutions utilized in NMR experiments. To ensure the reliability of the NOESY NMR data, which reflects interatomic distances, it was critical to confirm that the concentration of homoduplexes was sufficiently low at the specific temperature of 13 °C when these measurements were conducted.

When analyzed independently, the temperature-dependent UV absorption and ^1^H NMR data cannot provide conclusive information. This is because both possible two-state models – one accounting for hairpin folding (unimolecular transition) and the other for duplex formation (bimolecular transition) – fit the experimental data equally well. Therefore, attempting to fit a complex model considering both types of folded structures (hairpins and homoduplexes) to the results of only one of the two methods did not lead to convergence.

**Fig. 6:**
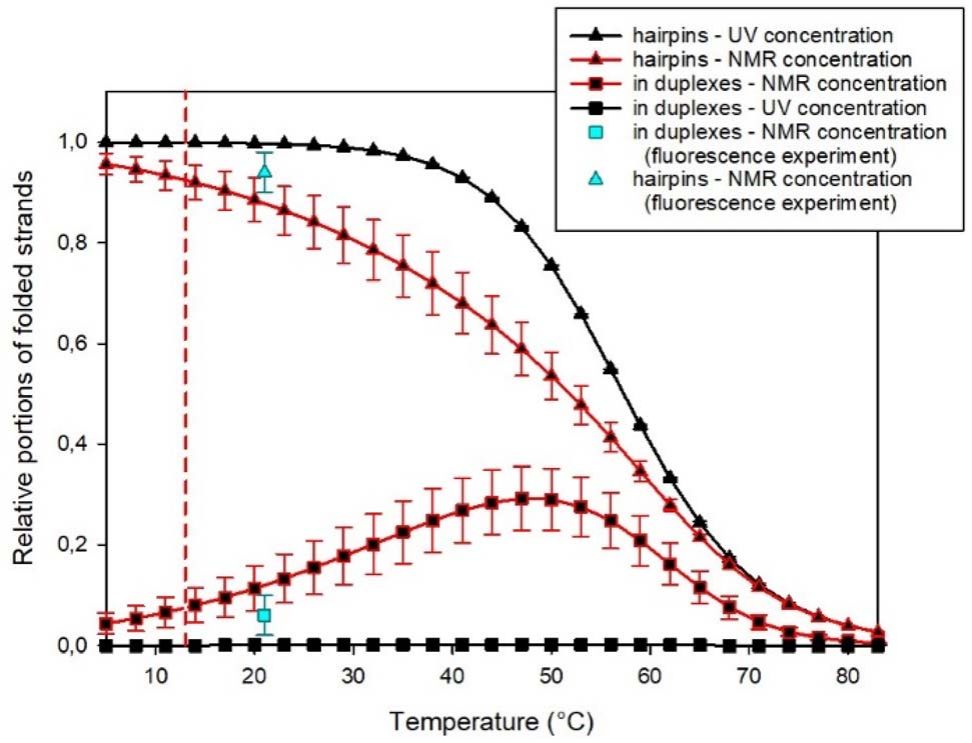
Estimated portions of SREseg16 strands in folded structures obtained from a simultaneous fit of UV and NMR data. Cyan symbols show estimates obtained from fluorescence experiments. The vertical dashed line indicates the temperature when NOESY NMR experiments were performed.

However, the substantial difference in the concentrations used in UV and NMR spectroscopies enabled simultaneous fitting by such a complex model to the results obtained from both experimental methods. In this way, we estimated the fraction of homoduplexes, as shown in Fig. 6 (see the ESI† for additional details about the procedure).

It becomes evident that at the high concentrations employed in NMR experiments, homoduplexes are indeed present, particularly around the temperature at which the hairpin structure melts. On the other hand, at the specific temperature of 13 °C, their estimated proportion is approximately (7 ± 3)% only.

This estimation was independently confirmed through a fluorescence experiment using SREseg16 oligonucleotides labeled with fluorescein (FAM) at the 3′ end and/or cyanine (Cy3) at the 5′ end. In this experiment, a fluorescence pattern comprising two excitation and two emission spectra was acquired and analyzed for each sample (see Method section for their list). Comparing the results obtained for double- and single-labeled cSREseg16 with those from a control double-labeled palindromic decamer, it became evident that the proximity of both fluorophores predominantly led to fluorescence quenching. Additionally, minor spectral changes were observed due to fluorescence resonant energy transfer.

To mitigate self-absorption effects, the concentration of fluorescently labeled oligonucleotides was kept at a maximum of 0.1 μM. To estimate the association constant for the homoduplex, a titration experiment was employed by mixing doubly labeled SREseg16 with an excess of unlabeled SREseg16. As an initial step, reference fluorescence patterns were established (a comprehensive description of the experiment and data treatment can be found in the ESI†). The titration of doubly labeled SREseg16 demonstrated that even with a significant excess of unlabeled SREseg16, only minimal changes in the fluorescence pattern occurred, even at the highest concentration ratio of 1 : 3000. Employing mathematical deconvolution based on the reference spectra, the association equilibrium constant for SREseg16 homoduplexes was determined to be (8 ± 6) μM^−1^. Consequently, the relative proportion of SREseg16 oligonucleotides in homoduplexes was estimated to be only (8 ± 5)% at the concentrations used in NMR measurements.

Moreover, it should be noted that the fluorescence experiments were conducted at ambient temperature (21 °C), whereas NOE measurements were performed at a lower temperature of 13 °C. Due to the decrease in homoduplex content at reduced temperatures (as illustrated in Fig. 6), the results from the fluorescence experiment indicated that the fraction of SREseg16 homoduplexes did not exceed 7% under the conditions of NOESY NMR measurements.

### Relation between the length and stability of the SREseg hairpin

**Fig. 7:**
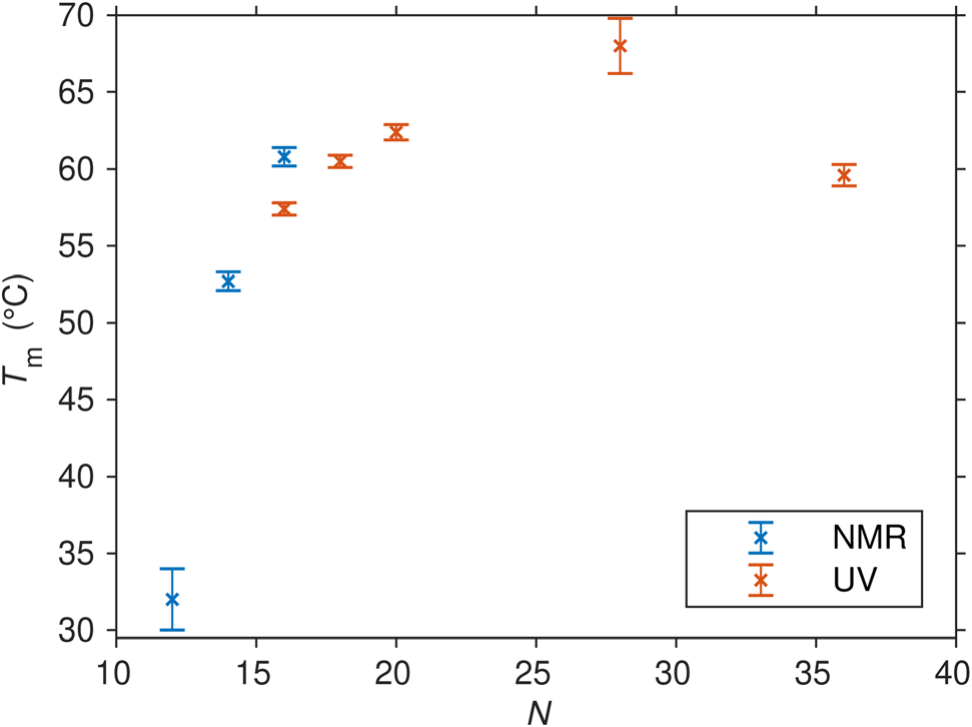
Melting points (and their uncertainties) of SREseg*N* oligomers folded into hairpins as determined from the fit of UV and NMR temperature-dependent data series.

Up to this point, our primary focus has been on SREseg16 as a model for the isolated folding behavior of a short oligonucleotide. However, it is crucial to acknowledge that SREseg16 constitutes only a small segment within the larger *c-Fos* gene sequence. To validate the general propensity for hairpin formation within this specific region and to ascertain that this behavior is not unique to 16-membered oligonucleotides, we extended our investigation to other SREseg*N* oligomers with lengths of *N* = {12, 14, 18, 20, 28, 36}. The obtained thermodynamic parameters characterizing the hairpin melting are summarized in Table 2, dependence of the melting temperature on the length of the used SRE segment is shown in Fig. 7. (Dependencies of the Δ*H* and Δ*S* are shown in Fig. S8 in the ESI.†).

The shorter ODNs, SREseg12 and SREseg14, were subjected to NMR analysis. When comparing SREseg16 to these shorter counterparts, specifically SREseg14, reducing the stem length by one base pair resulted in only a minor decrease in overall structure stability. The melting point of SREseg14 was found to be merely 8 °C lower than that of SREseg16. However, upon further reduction of the stem to just three base pairs (with three remaining unpaired nucleotides in the loop), the dynamic behavior of the hairpin loop becomes predominant, leading to a significant reduction in overall structure stability, as evidenced by a notable decrease in the melting point to 32 °C for SREseg12.

For the longer ODNs, melting points were determined by assessing and fitting the temperature-dependent UV absorption spectra. We employed the same procedure mentioned above for SREseg16, *i.e.*, SVD followed by simultaneous fit of relevant scores.§§Because of increasing melting temperature and thus shortening the temperature region corresponding to unfolded strands, the number of relevant scores (applicable factor dimension) was reduced from 4 to 3 for SREseg20 and longer oligonucleotides. Up to a length of *N* = 20, the hairpin stem extends to a maximum of seven WC base pairs, leading to a gradual increase in overall hairpin stability and a corresponding rise in the melting point. Beyond this point, the additional increase in stability becomes unnecessary, as SREseg16 already forms a sufficiently stable hairpin. Remarkably, a further enhancement in melting point is observed for SREseg28, even though the ODN ends are no longer complementary to each other (see Fig. 1) and thus cannot form WC base pairs within the stem. This increase in stability may be attributed to factors such as (i) stacking interactions with the bases forming the stem, (ii) additional non-canonical hydrogen bonds, or most likely, (iii) a combination of these effects. The longest ODN studied, SREseg36, already exhibits a slight decrease in stability compared to SREseg20 and SREseg28 due to a greater number of unpaired nucleobases at the ends of the hairpin (specifically 8). The flexibility of these ends contributes to reducing the stability of the hairpin.

In all cases, our observations indicate that hairpin formation within the CArG-box region on the *sense* strand of the *c-Fos* gene remains consistent regardless of the ODN length. Moreover, bases beyond the G-10 to C10 range may participate in the formation of the cruciform, involving an opposite hairpin on the *antisense* strand. This dynamic equilibrium between the double helix and the hairpin structure can be influenced by negative superhelical stress,^10,71,72^ which is readily induced during the early stages of transcription.

Numerous published studies have delved into DNA hairpin stability, structure, and dynamics using various experimental techniques (such as X-ray diffraction, UV absorption, calorimetry, gel electrophoresis, NMR, and fluorescence energy transfer) and computer simulations. However, these studies have predominantly focused on (i) short triloop and tetraloop hairpin structures,^68,73–78^ or (ii) DNA hairpins with exceedingly long loops (often consisting of only one type of nucleotide), thereby excluding consideration of stabilizing interactions within the loop.^76,79–81^ Consequently, some semiempirical rules have been established to predict the stability of hairpin DNA, accounting for loop parameters and the type of closing base pair.^82–85^ Nevertheless, the DNA hairpin structure discussed in this study, featuring a six-member loop and relatively high temperature stability, represents an unprecedented structural motif.

### Structure of the SREseg hairpin

The notably elevated melting point of the SREseg16 hairpin suggests the presence of additional stabilizing interactions beyond the Watson–Crick (WC) base pairing within the stem. This observation is reinforced by Fig. S9 in the ESI,† which displays the melting points of individual SREseg16 hydrogens. Remarkably, strong melting cooperativity extends across the entire molecule: the melting points derived from resonances of H6 and H8 on loop bases (A-3, T-2, A-1, T1, T2, and A3) closely resemble those of the nucleotides composing the stem, with differences within a mere 1 °C range. This similarity implies that the loop region is highly structured, compact, and exhibits limited flexibility, analogous to the stem.

**Fig. 8:**
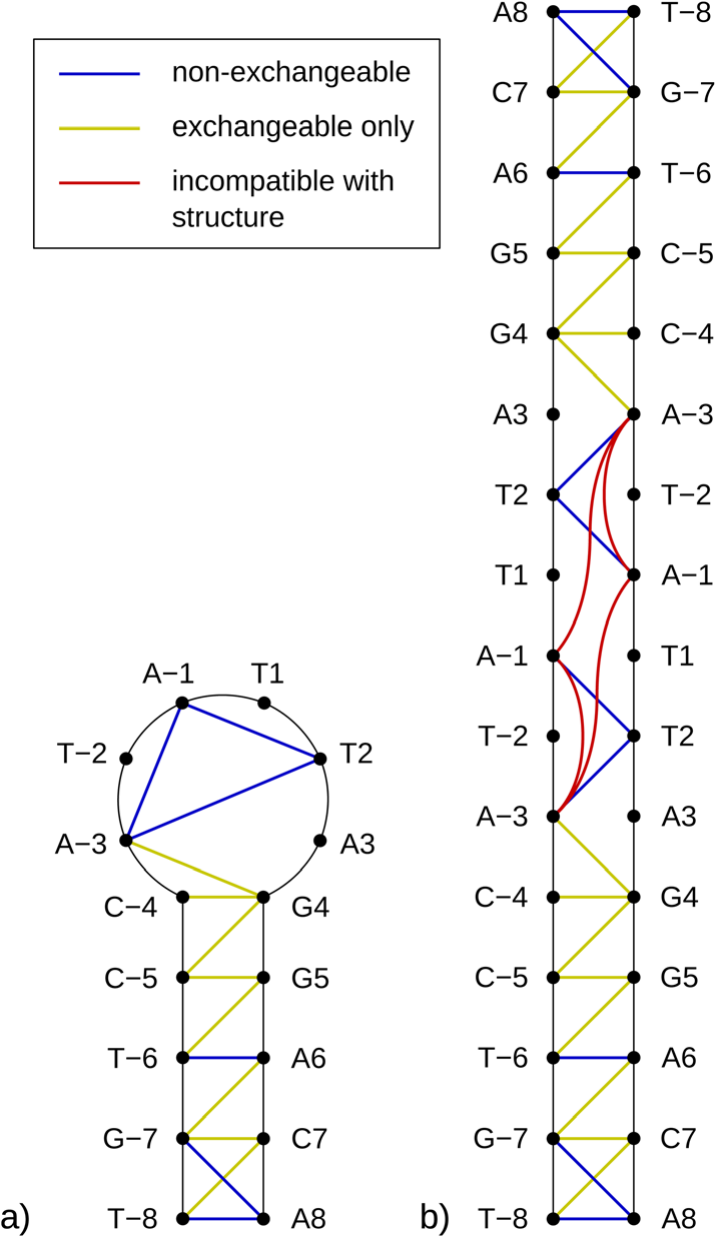
Diagram of connectivities in SREseg16 possible folds observed in the ^1^H NOESY spectra. Only NOE contacts between hydrogen nuclei from nonadjacent nucleosides are shown. (a) Hairpin; (b) mismatched duplex.

In the context of a standard sequential walk along the H6/H8 and H1′ resonances in the NOESY spectrum, the inter-nucleotide cross-peaks in the central segment, specifically those involving nucleosides between C-4 and A3 inclusive, exhibit lower intensities (Fig. 3). On average, the inter- and intranucleotide H1′–H6/H8 distances in the outer segment of SREseg16 closely align with the dimensions of regular B-DNA (described by ref. 86). However, in the central segment, the intranucleotide distances are shortened, while the internucleotide distances are increased. The NOESY spectrum reveals several cross-peaks that suggest an unconventional structure in the central portion of the sequence. Fig. 8 schematically illustrates the identified NOE contacts between non-adjacent nucleosides in SREseg16.

To gain deeper insights into these atomic-level stabilizing interactions, we constructed a model of the SREseg16 hairpin structure using computer modeling. Throughout molecular dynamics (MD) simulations, we applied constraints on the distances between selected atoms based on our NMR data, as summarized in Tables S1 and S2 in the ESI.†

**Fig. 9:**
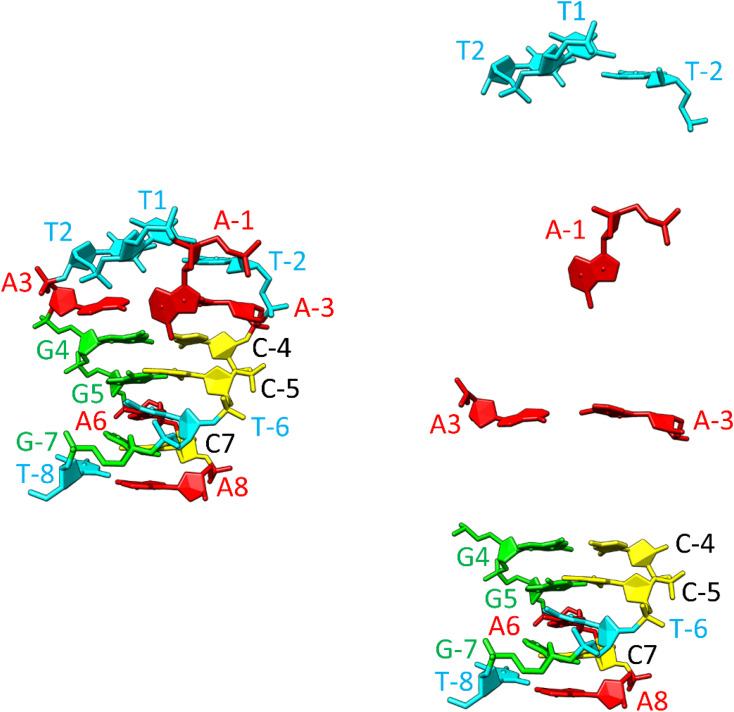
Structure of the SREseg16 hairpin (left – whole hairpin, right – from the bottom stem, atypical base pair AA_3, non-stacking A-1, and thymidine loop) resulting from the MD simulation. The nucleotide numbering corresponds to Fig. 1. The SREseg16 hairpin represents a highly compact fold stabilized not only by standard WC base-pairing in the stem, but also by extensive stacking (especially in the 3′ half), additional non-canonical inter-base hydrogen bonding (connecting A3 and A-3), and hydrophobic crowding (T2, T1, and T-2).

The resulting hairpin structure, as depicted in Fig. 9 (left), can be conceptually divided into stem and loop segments, as illustrated in Fig. 9 (right). The whole structure is stabilized through extensive stacking including the nucleotides in the loop. The stem is composed of five canonical WC base pairs, specifically AT_8, CG_7, AT_6, GC_5, and GC_4. The loop begins with an atypical AA_3 base pair, which is stabilized by one hydrogen bond (see Fig. S10, middle, in the ESI† for a different perspective). Additionally, A-1, the only non-stacking nucleotide, is attached to this base pair from the side. The remaining three thymidines (T2, T1, and T-2) form a compact loop, partially stabilized by the hydrophobic interactions resulting from the crowding of their methyl groups (Fig. S10, top, in the ESI†).

It is important to emphasize that the interactions involving T2 with A-1 and A-3 are unequivocal and directly supported by our NMR data (*i.e.*, T2 methyl group hydrogens – >A-3 H2, A-1 H8, A-1 H1′, A-1 H4′ as depicted in Fig. S10, bottom, and listed in Table S1 in the ESI†). Notably, these three nucleotides are not in immediate sequence proximity. In contrast, all other NMR data exclusively arise from interactions among immediately adjacent or opposing deoxynucleotides (Table S1 in the ESI†).

In summary, the SREseg16 hairpin structure represents a highly specific fold, characterized by stabilization mechanisms that encompass not only standard WC base-pairing within the stem but also extensive stacking, additional non-canonical inter-base hydrogen bonding (observed in the loop and the A3–A-3 base pair), and the influence of hydrophobic interactions arising from spatial crowding.

### Relevance to the SRF–SRE interaction

**Fig. 10:**
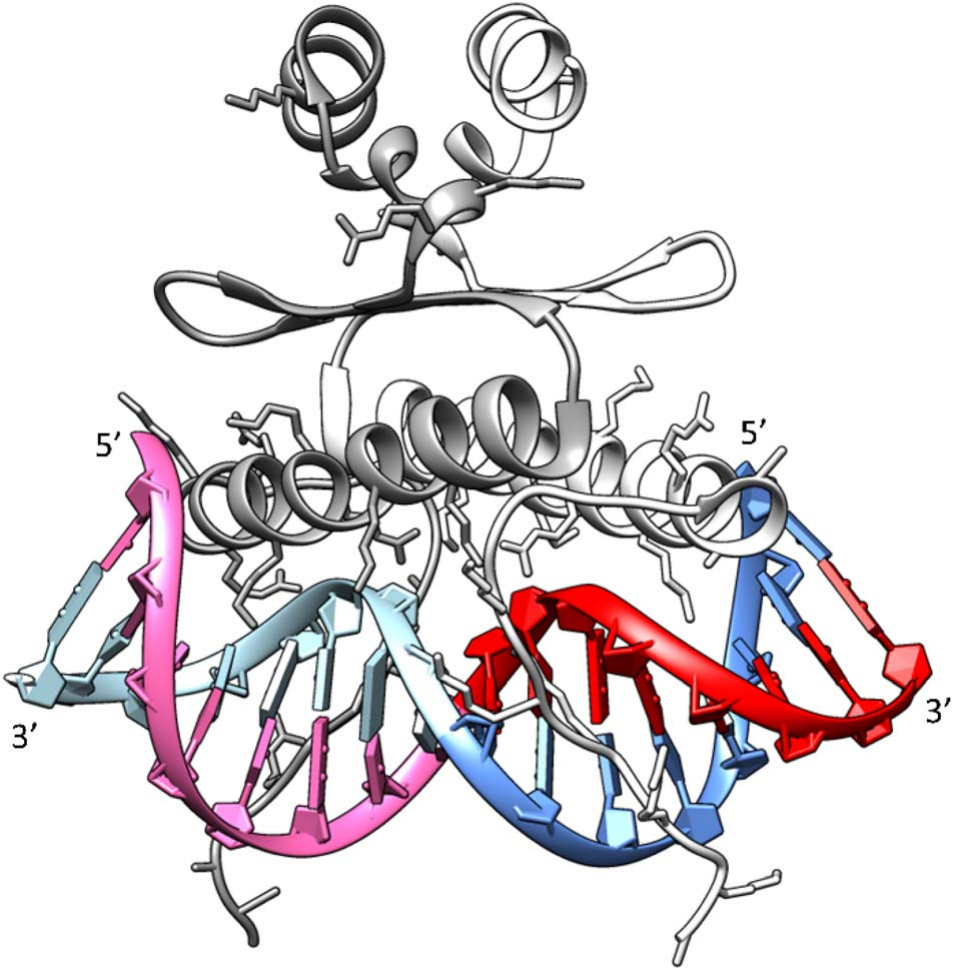
Structure of the SRF dimer complexed with the SRE DNA duplex. Adapted from ref. 39, PDB ID: 1k6o. Two SRF molecules are depicted in dark and light gray. DNA residues with positive numbering, as per Fig. 1, on the *sense* SRE strand are indicated in red, while negative numbered residues are represented in violet. Positive numbered residues of the *antisense* SRE strand are shown in cyan, and negative ones are presented in blue.

Drawing upon the established X-ray structure of the SRF dimer bound to the SRE segment of the *c-Fos* gene (shown in Fig. 10),^38,39^ we can postulate the potential significance of SRE hairpin and cruciform structures. Within the structure of SRF-SRE complex in Fig. 10, our hairpin forming SREseg sequence is colored in red (positively numbered nucleotides, T1 to A8) and violet (negatively numbered nucleotides, A-1 to T-8). The complementary sequence is colored in dark and light blue.

The formation of a cruciform, induced by DNA supercoiling during the initial stages of transcription, could expedite the rapid localization and recognition of the target SRF binding site due to topological considerations. In this scenario, one hairpin would be formed by interaction of red- and violet-colored nucleotides and second hairpin by dark a light blue ones. However, following the successful recognition, one of hairpins would remain independent of direct contact with SRF. Consequently, in a hypothetical situation where the cruciform SRE structure exists *in vivo*, its interaction with SRF may facilitate the reverse transition from a cruciform to a duplex. This transition would allow the SRE segment to engage with SRF through both DNA strands, leading to an increased number of salt bridges between positively charged amino acids (Arg and Lys) from SRF and the negatively charged sugar-phosphate backbone of SRE.

This enhanced interaction through a double-helical arrangement of the SRE–SRF complex would undoubtedly yield greater thermodynamic stability. The stimulation of the SRE transition from a cruciform to a duplex could, in theory, promote the initiation of transcription. However, to substantiate these hypotheses, it is imperative to conduct further *in vitro* investigations to assess the capacity of the second DNA strand within the SRE segment to form a hairpin and ultimately evaluate the potential of both DNA chains to adopt a cruciform motif.

There has been rapid development in this area recently. DNA cruciforms have been detected in growing mouse oocytes through immunofluorescence.^87^ Further, the kethoxal-assisted ssDNA sequencing^88,89^ revealed a strong correlation between ssDNA-containing regions and repetitive regions in the genome associated with alternative DNA structures, including Z-DNA (257 occurrences), G-quadruplexes (356 occurrences), hairpin structures (715 occurrences), triplex H-DNA (730 occurrences), and cruciforms (1643 occurrences). Moreover, S1-END-seq (where ssDNA-specific S1 nuclease opens hairpins and cleaves diagonally at the four-way junction of a cruciform to generate two-ended double-strand breaks and DNA ends are then captured by END-seq) reveals non-B-DNA secondary structures including cruciforms *in vivo* in expanded (TA)_*n*_^90^ that accumulate in microsatellite unstable human cancer cell lines.^91^ We believe that if the detailed results of such studies become publicly available, it will be possible to easily identify specific cruciforms in them. Subsequently, it will be possible to solve their structure *in vitro* through a combination of methods that have proven themselves in this study.

## Conclusions

Our study successfully demonstrated the *in vitro* formation of an exceptionally stable hairpin structure within the coding strand of the Serum Response Element of the *c-Fos* Gene Promoter (c-Fos SRE) through a combination of UV absorption and ^1^H NMR measurements. As anticipated, the stability of the hairpin amplifies with an augmented count of stem-forming complementary nucleotides bordering the central six-membered A/T tract. Remarkably, our findings reveal that three base pairs are sufficient for establishing a stable hairpin configuration at ambient temperature.

Across multiple sequences with 12 to 36 nucleotides centered around the CArG-box, the formation of the hairpin remained consistent. Notably, the stability of the hairpin persisted even in scenarios where an additional flanking strand (consisting of 8 nucleotides) was present at each end of the hairpin.

Our integrated analysis of UV, NMR, and complementary fluorescent experiments enabled precise determination of the relative abundance of mismatched homoduplexes across various temperature gradients. At the temperature of our NOESY experiment, the presence of homoduplexes was minimal (less than 7% for SREseg16), affirming the unambiguous assignment of the estimated interatomic distances.

Restrained molecular simulations, based on NMR-derived data for a sixteen-membered oligonucleotide, unveiled a highly compact hairpin structure fortified by extensive stacking. The rigid loop, constituted by the six-membered A/T sequence, showcased stability through non-canonical inter-base hydrogen bonding and hydrophobic packing of thymine methyl groups.

The strong evidence of a stable hairpin fold within the *c-Fos* SRE sequence supports the hypothesis that the *c-Fos* gene promoter can form a cruciform at the SRE site. This could facilitate its rapid recognition by transcription factor SRF. The possible hairpin formation by the complementary *c-Fos* SRE strand, its structural attributes, and the potential *in vitro* as well as *in vivo* formation of a cruciform structure by both SRE strands attract further exploration.

## Abbreviations

A/G/T/C: Adenine/guanine/thymine/cytosine
bp: Base pair
CArG-box: CC-AT rich-GG-box
DNA: Deoxyribonucleic acid
FA: Factor analysis
HMBC: Heteronuclear multiple bond correlation
IEG: Intermediate early gene
MD: Molecular dynamics
NOE: Nuclear Overhauser effect
NOESY: NOE spectroscopy
NMR: Nuclear magnetic resonance
ODN: Oligodeoxynucleotide
SRE: Serum response element
SRF: Serum response factor
SVD: Singular value decomposition
TCF: Ternary complex factor
UV/vis: Ultraviolet/visual
VT: Variable-temperature
WC: Watson–Crick

## Data availability

Most of the data is contained directly in the manuscript or in the Supplementary data (ESI†). Additional data (mainly for shorter and longer SREseg*N* oligodeoxynucleotides) can be shared upon request. If you are interested, contact the corresponding author.

## Author contributions

Barbora Profantová: data curation, formal analysis, investigation. Václav Římal: formal analysis, validation, investigation, visualization, methodology, writing-original draft. Václav Profant: formal analysis, validation, visualization, methodology, writing-original draft, writing-review and editing. Ondřej Socha: data curation, formal analysis. Ivan Barvík: data curation, formal analysis, validation, investigation, visualization, methodology, writing-original draft. Helena Štěpánková: methodology. Josef Štěpánek: conceptualization, data curation, formal analysis, validation, investigation, visualization, methodology, writing-original draft, supervision.

## Conflicts of interest

The authors declare that they have no conflicts of interest with the contents of this article.

## Supplementary Material

RA-014-D4RA05897F-s001
