# Supplementary material for: Polymorphic potential of SRF binding site of c-Fos gene promoter: in vitro study
Source: RSC Adv. 2024 Dec 3;14(51):38253–67. doi: 10.1039/d4ra05897f (PMC11613138; doi:10.1039/d4ra05897f)
Supplement: RA-014-D4RA05897F-s001 [file RA-014-D4RA05897F-s001.pdf]

Supplementary Information (SI) for RSC Advances.
This journal is © The Royal Society of Chemistry 2024

Polymorphic potential of SRF binding site of *c-Fos* Gene Promoter: *In vitro* study

Barbora Profantová,[1] Václav Římal,[2] Václav Profant,[1]* Ondřej Socha,[2#] Ivan Barvík,[1] Helena Štěpánková,[2] Josef Štěpánek[1]

From the [1]Institute of Physics, Faculty of Mathematics and Physics, Charles University, Ke Karlovu 5, 121 16 Prague 2, Czech Republic; [2]Department of Low-Temperature Physics, Faculty of Mathematics and Physics, Charles University, V Holešovičkách 2, 180 00 Prague 8, Czech Republic

[#]Present address: Institute of Organic Chemistry and Biochemistry, Academy of Sciences, Flemingovo náměstí 2, 166 10 Prague 6, Czech Republic

Content:

- **Fitting equations**

- **Table S1** Inter-hydrogen distances in SREseg16 obtained based on NOESY (non-exchangeable protons).
- **Table S2** Inter-hydrogen distances in SREseg16 obtained based on NOESY (exchangeable protons).

- **Figure S1** UV absorption spectra of SREseg16.
- **Figure S2** Additional spectral components from SVD of SREseg16 UV absorption temperature dependence.
- **Figure S3** ^{1}H–^{13}C HMBC spectrum with assignment of adenine H2 resonances by their links to cross peaks between H8 and C4.
- **Figure S4** ^{1}H NMR spectra (part of the region of nonexchangeable base protons) of SREseg16 (left) in comparison with a duplex forming palindromic CTTGCAAG sequence (right) measured at various temperatures.
- **Figure S5** FWHM of ^{1}H resonances at temperatures with stable hairpin SREseg16 and the reference duplex before the melting starts influencing the line widths.
- **Figure S6** Correlations between predicted and experimental chemical shifts in the folded state (298 K) for the stem and the loop.
- **Figure S7** Slopes of the chemical shifts with respect to the temperature of SREseg16 in the folded and unfolded state.
- **Figure S8** Thermodynamic parameters ΔH and ΔS of SREseg*N* oligomers folded into hairpins as determined from the fit of UV and NMR temperature-dependent data series.
- **Figure S9** Melting temperatures observed for individual ^{1}H resonances in NMR spectra of SREseg16.
- **Figure S10** Details of the SREseg16 hairpin loop structure resulting from MD simulation.

- **Common fit of UV and NMR data to a complex model** (formation of both hairpins and homoduplexes)

- **Fluorescence experiments** (estimation of homoduplexes present in highly concentrated solutions of SREseg16)

Fitting equations

Although the same thermochemistry models (see the Experimental procedures section for details) were used to evaluate UV absorption measurements and NMR experiments, the specific shape of the fitting equations is different in both cases. In the case of UV absorption, we perform a joint fit of the course of V_1-V_M scores obtained by SVD. The merit function to be minimized by the fit of hairpin melting is

$$\sum_{i=1}^{n}\sum_{j=1}^{M}\left[\sqrt{W_j}V_{ij}-\left(a_{j0}+a_{j1}T_i\right)c_i^{\mathrm{h}}-\left(b_{j0}+b_{j1}T_i\right)c_i^{\mathrm{un}}\right]^2=min, \qquad \text{(S1)}$$

while the equation for duplex melting is

$$\sum_{i=1}^{n}\sum_{j=1}^{M}\left[\sqrt{W_j}V_{ij}-\left(a_{j0}+a_{j1}T_i\right)2c_i^{\mathrm{du}}-\left(b_{j0}+b_{j1}T_i\right)c_i^{\mathrm{un}}\right]^2=min. \qquad \text{(S2)}$$

where n is the number of spectra measured in the temperature dependence and M is an applicable factor dimension (equal to 4 or 3 depending on the length of oligonucleotides).

In the case of variable-temperature ^{1}H NMR experiments, we perform a fit of chemical shifts of individual hydrogens depending on temperature. The fitting equation for melting of a hairpin is

$$\delta(T)=(a_0+a_1T)\,c_{\mathrm{h}}/c+(b_0+b_1T)\,c_{\mathrm{un}}/c\ , \qquad \text{(S3)}$$

while the equation for melting of a duplex is

$$\delta(T)=(a_0+a_1T)\,2c_{\mathrm{du}}/c+(b_0+b_1T)\,c_{\mathrm{un}}/c\ . \qquad \text{(S4)}$$

Table S1 Inter-hydrogen distances in SREseg16 obtained from fitting and integration of cross-peaks of non-exchangeable protons in NOESY spectrum recorded at 13 °C.

Assignment	V (a.u.)	σ_V (a.u.)	L (Å)	σ_L (Å)
T-8 H1' ⋯ T-8 H6	81	36	3.3	0.2
T-8 H1' ⋯ G-7 H8	20	4	4.2	0.1
T-8 H1' ⋯ A8 H2	18	4	4.2	0.1
T-8 H1' ⋯ T-8 M7	95	19	3.2	0.1
G-7 H1' ⋯ G-7 H8	49	24	3.6	0.3
G-7 H1' ⋯ T-6 H6	112	25	3.1	0.1
G-7 H1' ⋯ T-6 M7	17	5	4.2	0.2
G-7 H1' ⋯ A8 H2	11	2	4.6	0.2
G-7 H8 ⋯ T-6 H6	29	5	3.9	0.1
G-7 H8 ⋯ T-6 M7	40	6	3.7	0.1
T-6 H1' ⋯ C-5 H6	71	13	3.4	0.1
T-6 H1' ⋯ A6 H2	41	50	3.7	0.7
T-6 H6 ⋯ T-6 M7	81	12	3.3	0.1
T-6 H6 ⋯ C-5 H5	37	7	3.7	0.1
T-6 M7 ⋯ C-5 H5	14	3	4.4	0.2
C-5 H1' ⋯ C-5 H6	78	6	3.29	0.04
C-5 H1' ⋯ C-4 H6	115	4	3.09	0.02
C-5 H5 ⋯ C-5 H6	640	260	2.3	0.2
C-4 H1' ⋯ A-3 H8	34	7	3.8	0.1
C-4 H5 ⋯ C-4 H6	500	160	2.4	0.1
C-4 H5 ⋯ A-3 H2	48	10	3.6	0.1
C-4 H6 ⋯ A-3 H8	29	21	3.9	0.5
A-3 H1' ⋯ A-3 H8	101	8	3.15	0.04
A-3 H1' ⋯ T-2 M7	6	4	5.1	0.6
A-3 H2 ⋯ T-2 M7	2.3	0.5	5.9	0.2
A-3 H2 ⋯ A-1 H1'	42	6	3.7	0.1
A-3 H2 ⋯ T2 M7	6	1	5.1	0.2
A-3 H8 ⋯ T-2 M7	9	2	4.7	0.2
T-2 H1' ⋯ T-2 H6	79	20	3.3	0.1
T-2 H1' ⋯ A-1 H8	5	1	5.2	0.2
T-2 H6 ⋯ T-2 M7	88	18	3.2	0.1
T-2 M7 ⋯ A-1 H2	5	1	5.2	0.2
A-1 H1' ⋯ A-1 H8	86	20	3.2	0.1
A-1 H1' ⋯ T1 M7	10	2	4.6	0.2
A-1 H1' ⋯ T2 M7	5	1	5.2	0.2
A-1 H4' ⋯ T2 M7	2.3	0.5	5.9	0.2
A-1 H8 ⋯ T1 M7	7	3	5.0	0.4
A-1 H8 ⋯ T2 M7	1.8	0.1	6.2	0.1

Assignment	V (a.u.)	σ_V (a.u.)	L (Å)	σ_L (Å)
T1 H1' ⋯ A-1 H2	19	4	4.2	0.1
T1 H1' ⋯ T1 H6	95	4	3.19	0.02
T1 H1' ⋯ T2 H6	5	1	5.1	0.2
T1 H1' ⋯ T2 M7	11	7	4.6	0.5
T1 H6 ⋯ A-1 H8	20	4	4.1	0.1
T1 H6 ⋯ T1 M7	70	11	3.4	0.1
T1 M7 ⋯ A-1 H2	2.8	0.6	5.7	0.2
T2 H1' ⋯ T2 H6	140	50	3.0	0.2
T2 H1' ⋯ T2 M7	9	2	4.7	0.2
T2 H6 ⋯ T2 M7	80	19	3.3	0.1
T2 M7 ⋯ T1 H6	14	3	4.4	0.2
T2 M7 ⋯ A3 H2	7.9	0.6	4.8	0.1
A3 H1' ⋯ A3 H8	73	15	3.3	0.1
A3 H1' ⋯ G4 H8	65	23	3.4	0.2
G4 H1' ⋯ G4 H8	50	18	3.5	0.2
G4 H1' ⋯ G5 H8	86	15	3.3	0.1
G5 H1' ⋯ G5 H8	52	19	3.5	0.2
G5 H1' ⋯ A6 H8	87	17	3.2	0.1
A6 H1' ⋯ A6 H8	60	3	3.45	0.03
A6 H1' ⋯ C7 H6	86	3	3.24	0.02
A6 H8 ⋯ C7 H6	49	10	3.6	0.1
C7 H1' ⋯ A6 H2	41	8	3.7	0.1
C7 H1' ⋯ A8 H8	49	10	3.6	0.1
C7 H5 ⋯ A6 H8	59	12	3.5	0.1
C7 H5 ⋯ C7 H6	320	100	2.6	0.1
C7 H6 ⋯ A8 H8	22	4	4.1	0.1
A8 H1' ⋯ A8 H2	18	4	4.2	0.1
A8 H1' ⋯ A8 H8	80	12	3.3	0.1

V – integrated NOESY cross-peak volume; L – calculated inter-hydrogen distance; σ_V and σ_L – standard errors of V and L, respectively

Table S2 Inter-hydrogen distances in SREseg16 obtained from fitting and integration of cross-peaks involving exchangeable protons in NOESY spectrum recorded at 13 °C.

**Assignment**	***V*** (a.u.)	σ_V (a.u.)	***L*** (Å)	σ_L (Å)
**G-7 H1 ··· A6 H2**	38	11	3.7	0.2
**G-7 H1 ··· A8 H2**	45	23	3.6	0.3
**G-7 H1 ··· C7 H41**	115	23	3.1	0.1
**G-7 H1 ··· C7 H42**	77	29	3.3	0.2
**G-7 H1 ··· T-6 H3**	20	4	4.1	0.1
**T-6 H3 ··· A6 H2**	130	33	3.0	0.1
**T-6 H3 ··· G5 H1**	23	5	4.1	0.2
**T-6 M7 ··· C-5 H41**	17	12	4.2	0.5
**T-6 M7 ··· C-5 H42**	21	5	4.1	0.2
**C-5 H5 ··· C-5 H42**	217	43	2.78	0.09
**C-5 H41 ··· G5 H1**	165	33	2.9	0.1
**C-5 H41 ··· C-5 H5**	109	41	3.1	0.2
**C-5 H41 ··· C-5 H42**	690	140	2.3	0.1
**C-5 H42 ··· C-4 H41**	3	3	6	1
**C-5 H42 ··· G4 H1**	94	19	3.2	0.1
**C-5 H42 ··· G5 H1**	106	36	3.1	0.2
**C-4 H41 ··· G4 H1**	118	24	3.1	0.1
**C-4 H41 ··· C-4 H5**	120	33	3.1	0.1
**C-4 H41 ··· C-4 H42**	560	110	2.4	0.1
**C-4 H42 ··· G4 H1**	72	17	3.3	0.1
**C-4 H42 ··· C-4 H5**	163	86	2.9	0.3
**G4 H1 ··· A-3 H2**	41	8	3.7	0.1
**G4 H1 ··· A3 H2**	21	4	4.1	0.1
**G5 H1 ··· A6 H2**	32	17	3.8	0.3
**C7 H5 ··· C7 H41**	135	27	3.0	0.1
**C7 H5 ··· C7 H42**	239	48	2.73	0.09
**C7 H41 ··· C7 H42**	610	120	2.34	0.08
**C7 H42 ··· T-8 M7**	10	2	4.66	0.2

V – integrated NOESY cross-peak volume; *L* – calculated inter-hydrogen distance; σ_V and σ_L – standard errors of *V* and *L*, respectively

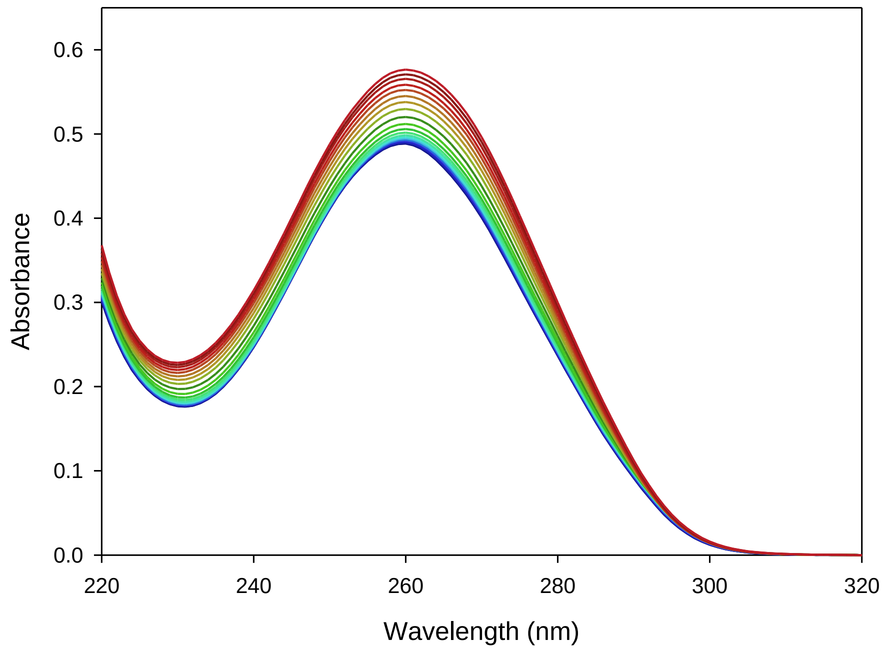

Figure S1. UV absorption spectra of SREseg16 measured at temperatures from 5 °C (blue line) – 85 °C (red line) with ~4 °C step.

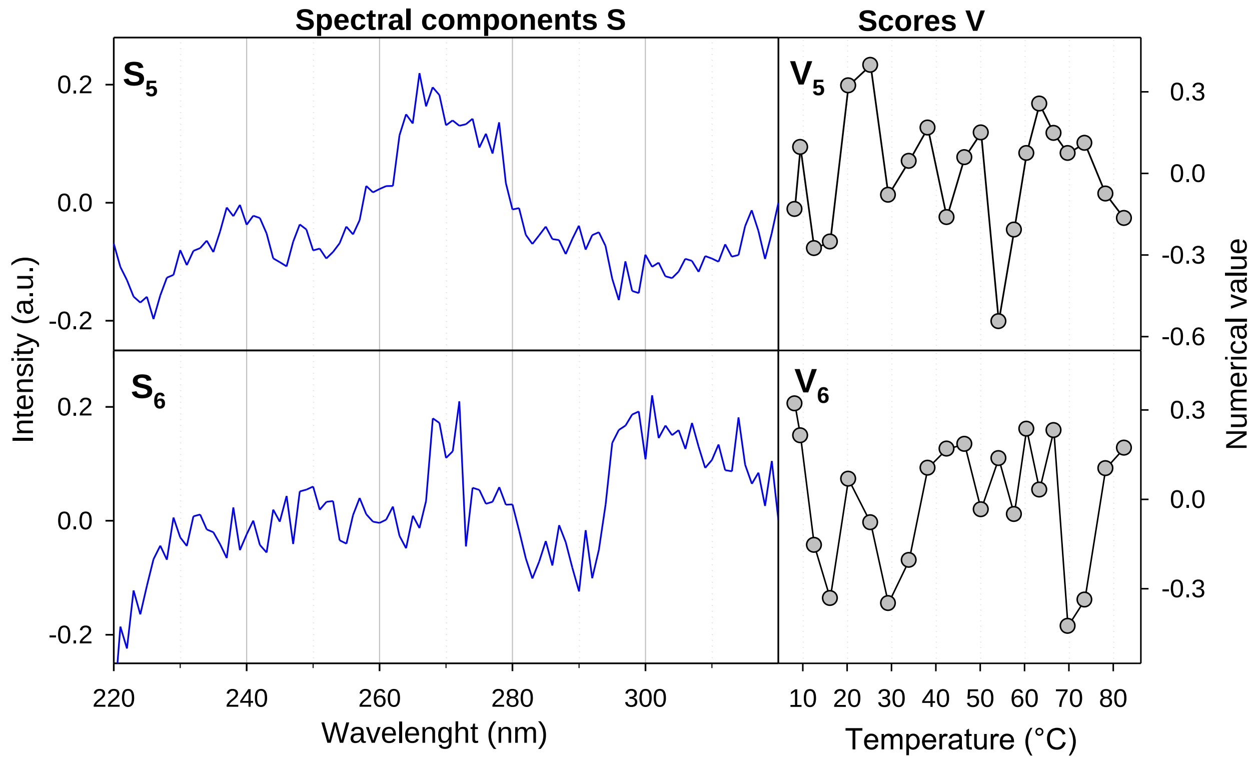

Figure S2. Additional spectral components from singular value decomposition of SREseg16 UV absorption temperature dependence spectral series. Random courses of spectral scores *V* confirm that analyzed spectral series have factor dimension less than five.

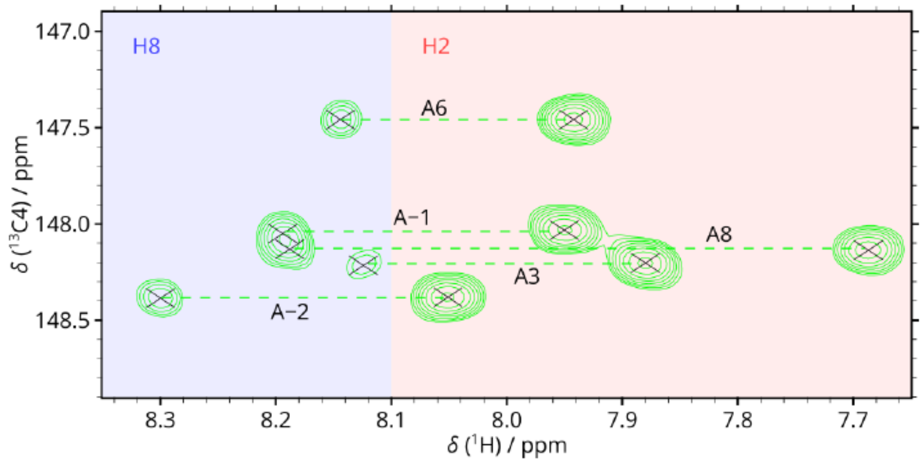

Figure S3. ^{1}H–^{13}C HMBC spectrum with assignment of adenine H2 resonances by their links (horizontal dashed lines labeled by nucleotide) to cross peaks between H8 and C4. Regions with ^{1}H chemical shifts of H8 and H2 are indicated by blue and red background, respectively.

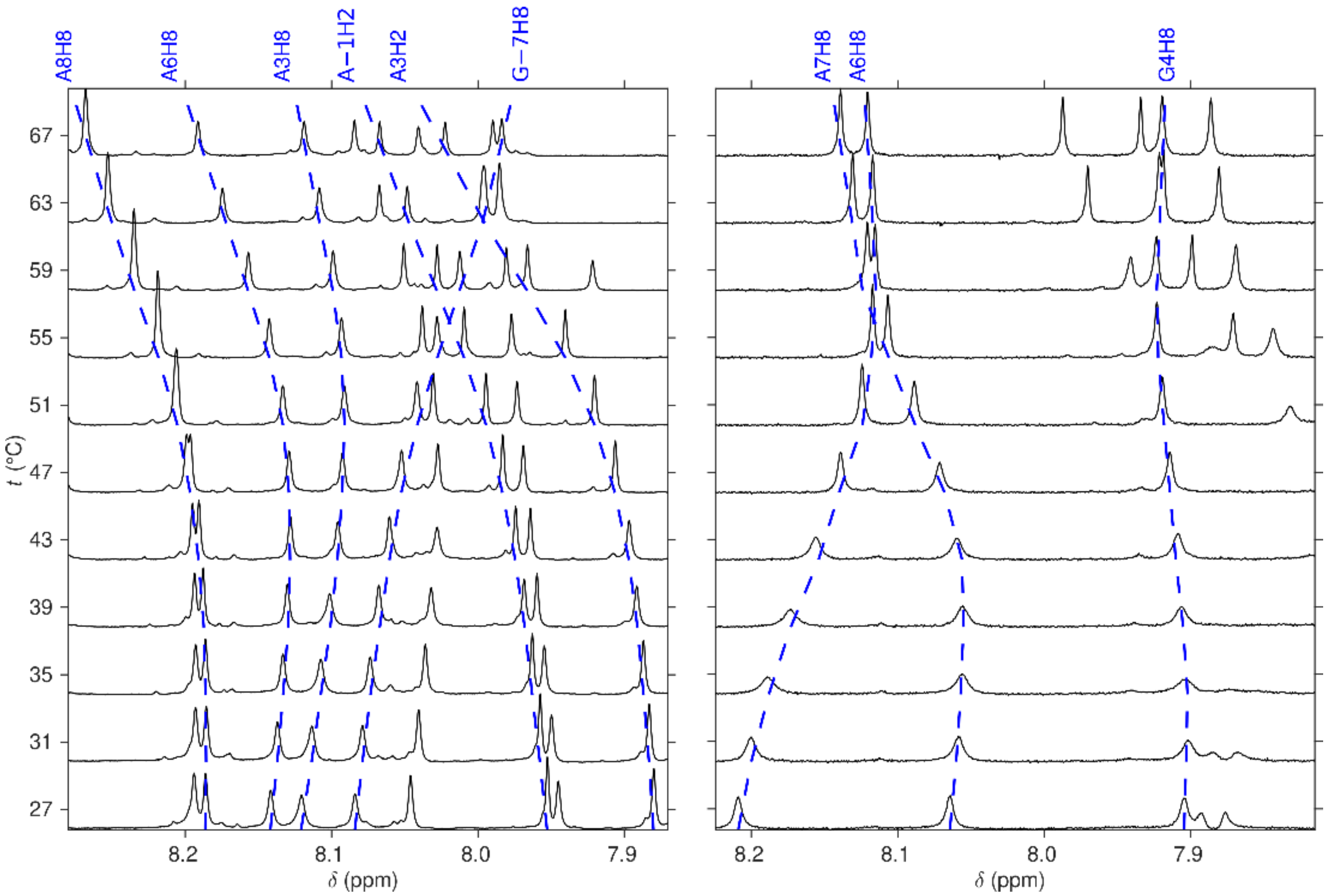

Figure S4. ^{1}H NMR spectra (part of the region of nonexchangeable base protons) of SREseg16 (left) in comparison with a duplex-forming palindromic CTTGCAAG sequence (right) measured at various temperatures *t*. The positions of selected resonances, labeled at the top, are tracked by the dashed lines.

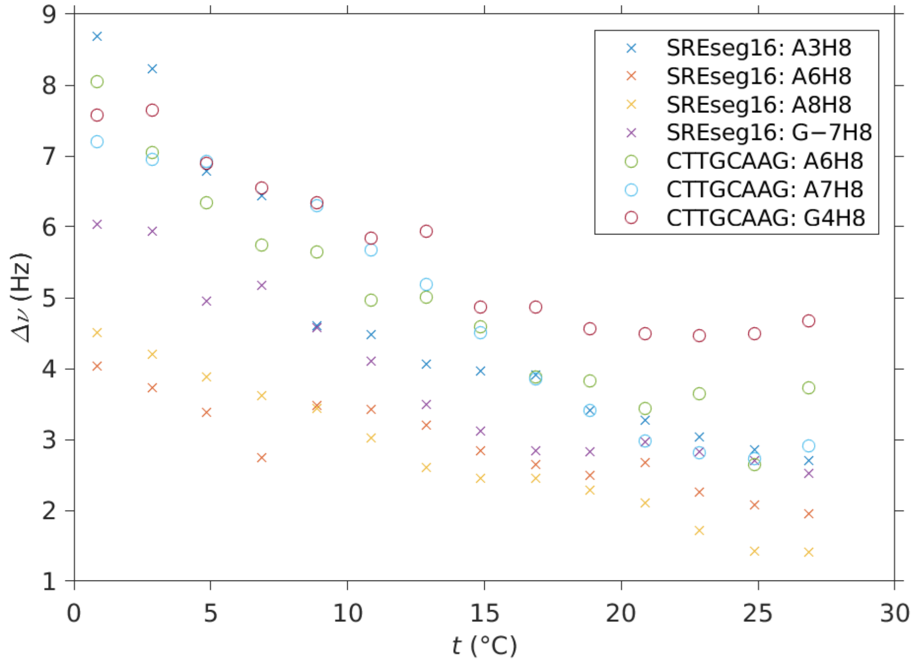

Figure S5. FWHM of ^{1}H resonances (shown in the spectra in Figure S4) at temperatures with stable hairpin SREseg16 (crosses) and the reference duplex $(CTTGCAAG)_2$ (circles) before the melting starts influencing the line widths.

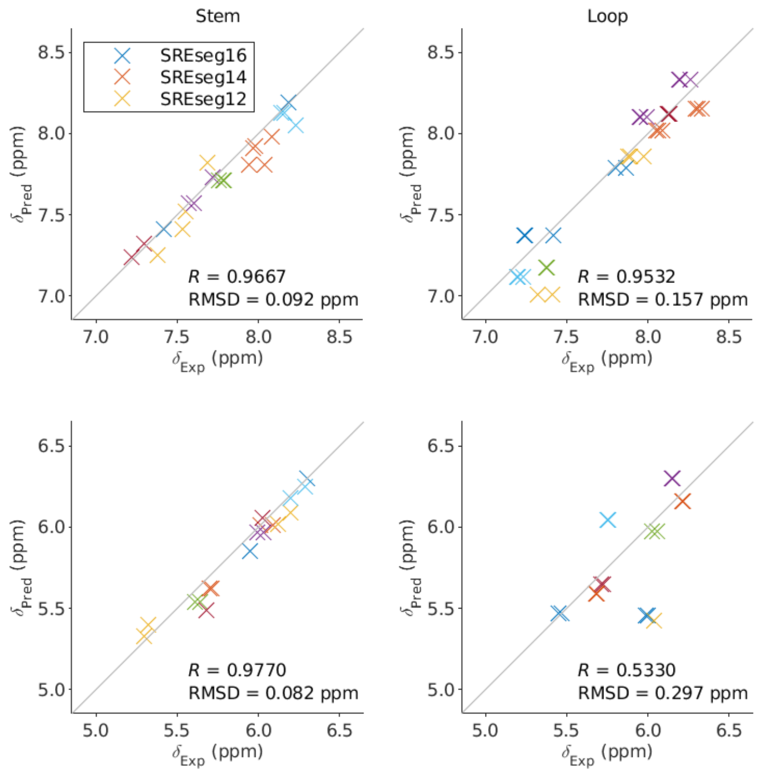

Figure S6. Correlations between predicted (for duplexes) and experimental chemical shifts in the folded state (298 K) for the stem excluding the last C·G pair (N±8–N±5, left) and the loop (N±4–N±1, right). Correlations were made for H6/H8/H2 (top) and H1'/H5 (bottom). Correlation coefficients *R* and root-mean-square-distances RMSD are shown. The predicted values are corrected for the presence of mismatch base pairs.

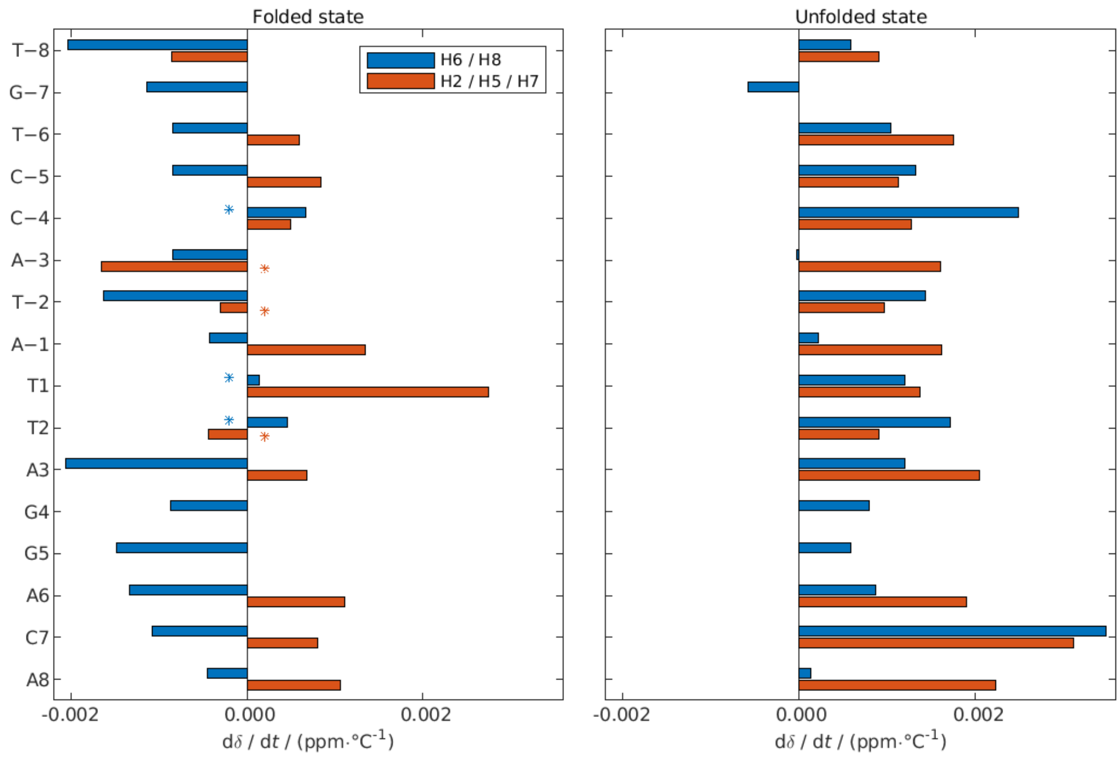

Figure S7. Slopes of the chemical shifts with respect to temperature in the linear regions of SREseg16 in the folded (left) and unfolded state (right). Asterisks mark opposite signs than those observed for self-complementary 8-bp DNA duplexes.

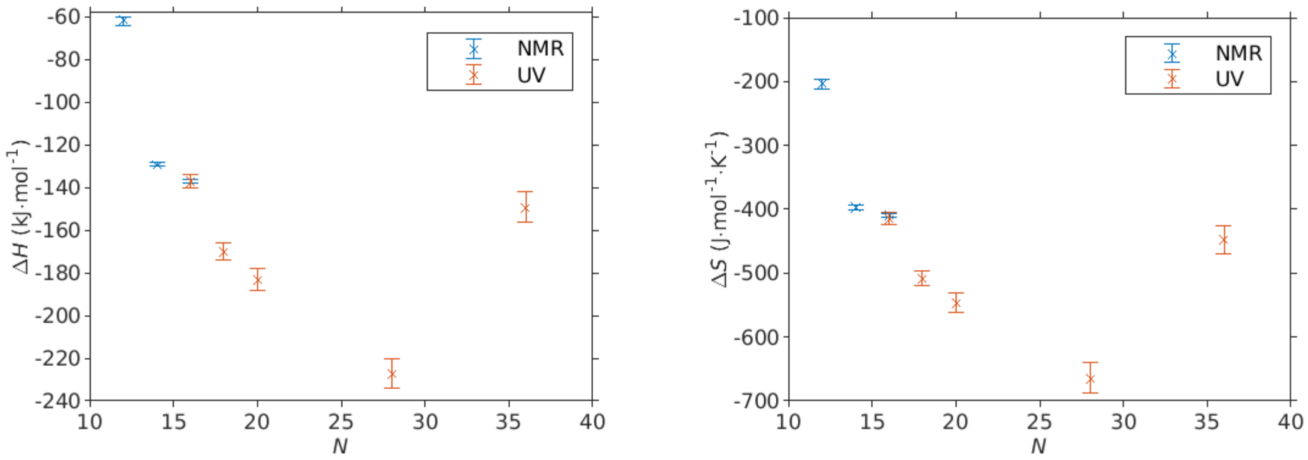

Figure S8. Thermodynamic parameters ΔH (left) and ΔS (right) and their uncertainties of SREseg*N* oligomers folded into hairpins as determined from the fit of UV and NMR temperature dependent data series.

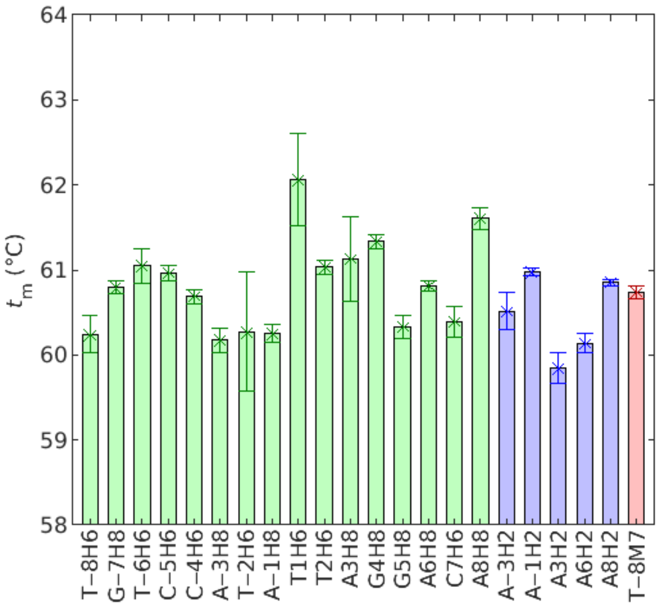

Figure S9. Melting temperatures, t_m, observed for individual aromatic ^{1}H resonances in NMR spectra of SREseg16.

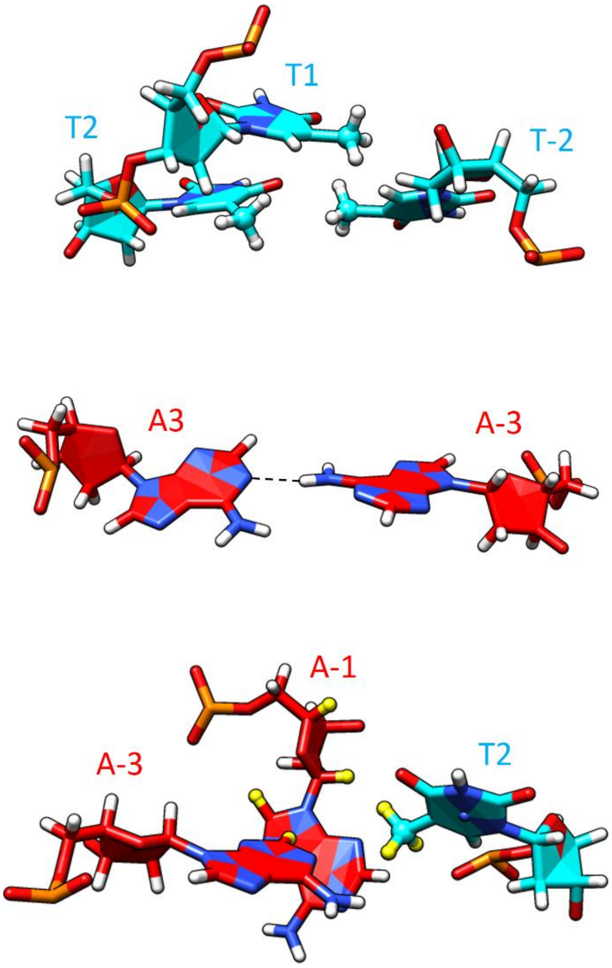

Figure S10. Details of the SREseg16 structure resulting from MD simulation. Bottom - Mutual orientation of A-3, A-1, and T2 nucleotides with highlighted hydrogen atoms (in yellow) whose spatial proximity was revealed in NOESY experiment (Table S1). Middle – Atypical AA_3 base pair stabilized by one hydrogen bond. Top – Hydrophobic crowding of three methyl groups originating from T2, T1, and T-2 stabilizing the central part of the loop.

Common fit of UV and NMR data to a complex model considering both formation of hairpins and homoduplexes

To couple the data from two different experiments, we based the common fit on the temperature dependences of the relative percentages of the unfolded form obtained from the previous fits of the two-state models. As these dependences were slightly different for the hairpin and the homoduplex model, we obtained two possible curves for each experiment (Figure SI E1, upper part). The common fit was then applied to the four possible combinations of the curves. The average values of the thermodynamic parameters and their standard deviations were first calculated for all the four combinations (Figure SI E1, bottom part).

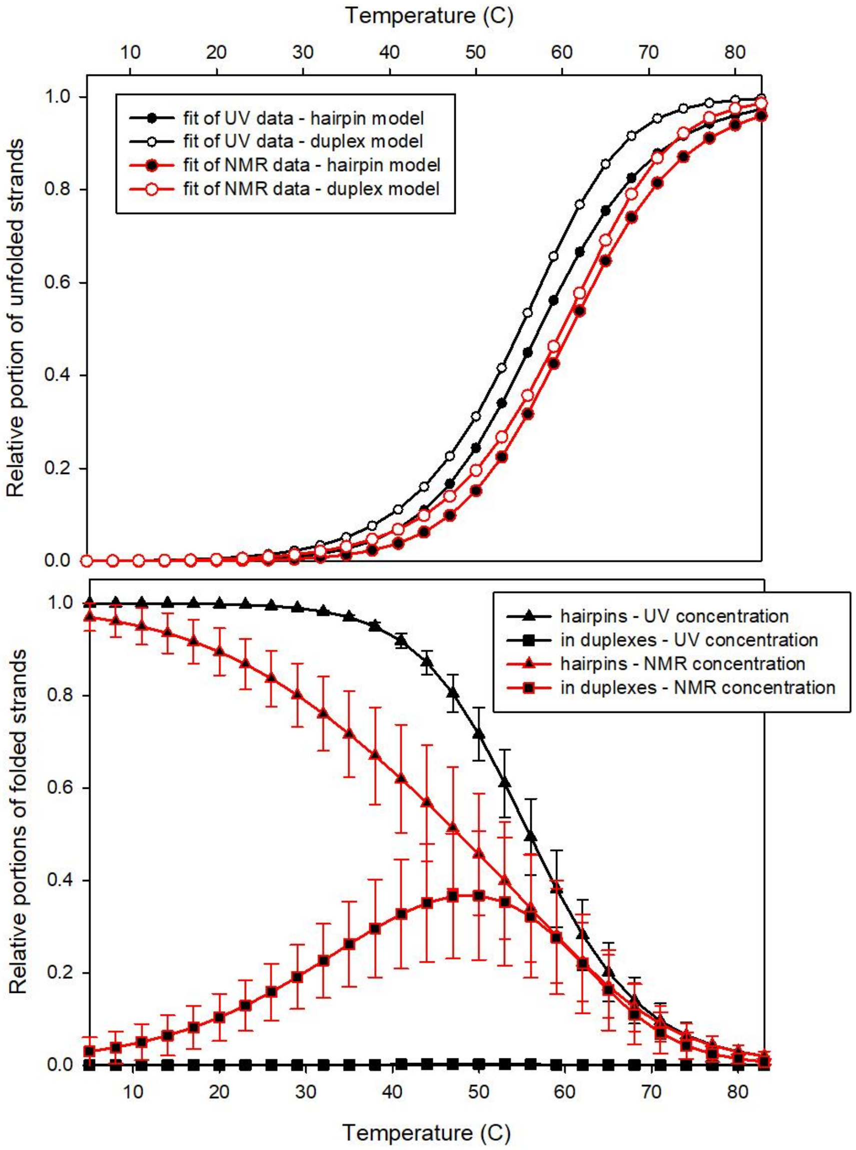

Fig. SI-E1. Top: Temperature dependences of unfolded SREfos16 strands obtained from UV and NMR experiment. Bottom: Temperature dependences of relative portions of the strands folded in hairpins and imperfect homoduplexes obtained by a fit of a complex model to the above shown data.

Because all the fits resulted in a zero homonucleotide presence for the UV concentration conditions, we after took into account only the fits combining the curve of the harpin model for the low concentration and the two both curves for the high concentration. The percentages of oligonucleotide strands in the hairpin, homoduplex and unfolded form are shown in Fig. 6. It follows that in the case of the high concentration used in NMR experiments, the homoduplexes are present mainly around the temperature of the hairpin melting, but at the temperature of 13C their portion is only (7±3) %.

Fluorescence experiments

(estimation of homoduplexes present in highly concentrated solutions of SREseg16)

Basic spectra

Spectra of double and single-labeled SREseg16 are shown in Figure SI-F1 together with a control of unlabeled oligonucleotide and double-labeled palindrome (Pal10). Practically zero fluorescence signal of unlabeled oligonucleotides demonstrates a lack of parasitic signals from impurities or badly filtered elastic scattering.

The spectra of double-labeled SREseg16 (forming dominantly hairpin) indicate that the proximity of the two fluorescence labels causes dominantly the fluorescence quenching even if there are also signs of the energy transfer (e.g., relative increase of emission at 560 nm when excited at 495 nm). In the case of the Pal10 duplex, the effect is enhanced by the presence of two FAM/Cy3 pairs in a short distance at opposite sides of a duplex (in agreement with the conclusions in *Dietrich 2002*.)

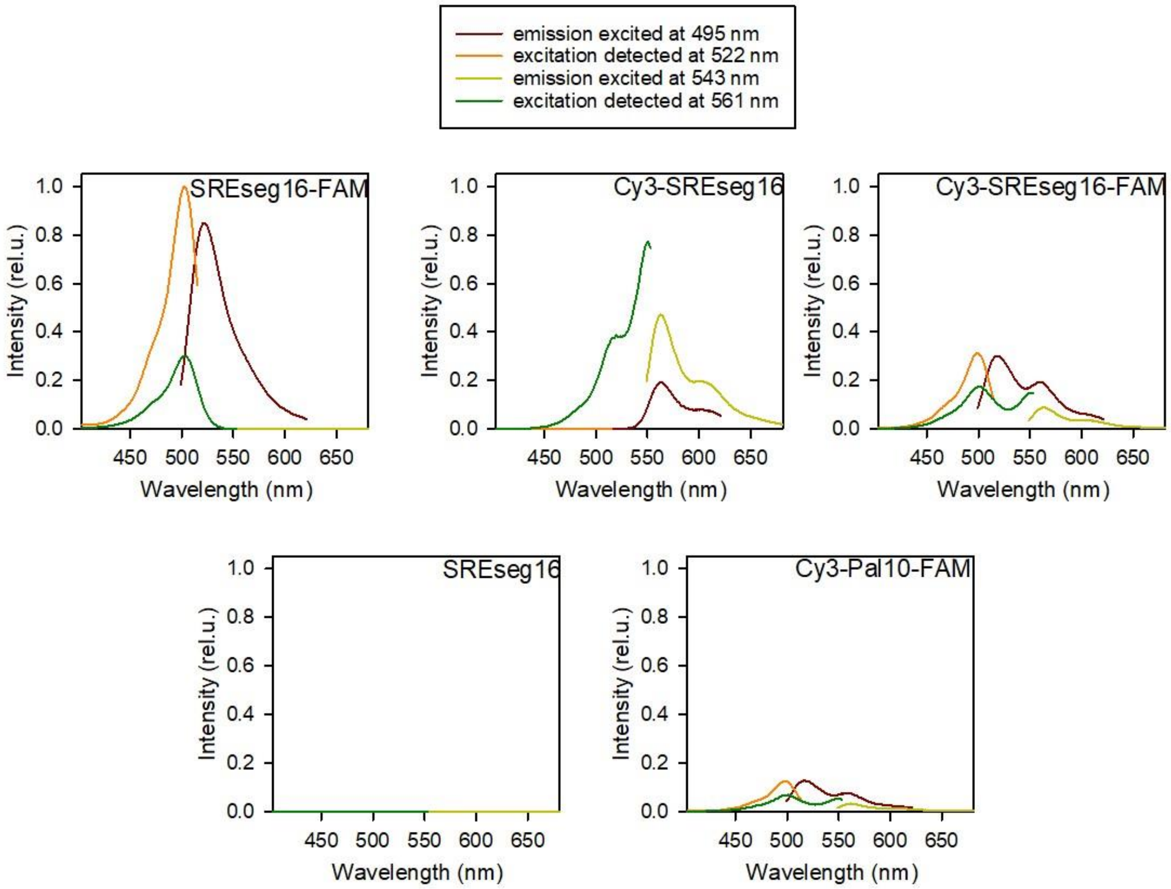

Figure SI-F1. Fluorescence spectra of 0.1µM solutions of single- and double-labeled SREseg16, unlabeled SREseg16, and double-labeled self-complementary decamer Pal10. The same intensity scale is fixed for all graphs in this figure as well as in Figures SI-F2, SI-F4, and SI-F5.

Mixtures of double-labeled and unlabeled strands

In mixtures of double-labeled and unlabeled strands, "hybrid" duplexes consisting of one double-labeled and one unlabeled strand are formed. In this case, the increased distance between FAM and Cy3 should cause a reduction of the fluorescence quenching and change energy-transfer effects with respect to hairpins. The advantage of these experiments is that the concentration of unlabeled oligonucleotide is not limited by its absorption. On the other hand, one can expect that the increase of FAM and Cy3 distance caused by the duplex formation will not be sufficient to change fluorescence spectra dramatically.

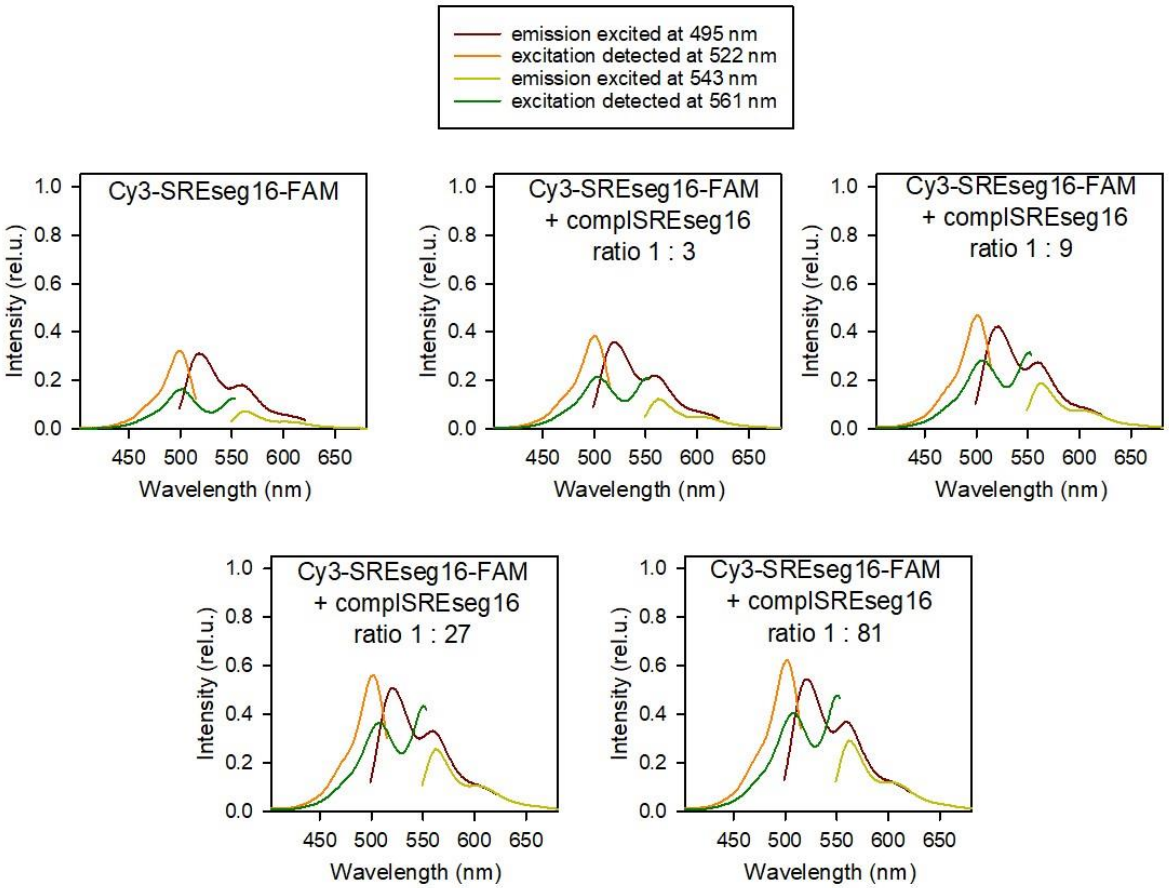

Figure SI F2. Fluorescence spectra of 0.1µM Cy3-SREseg16-FAM with excess of unlabeled complementary strand.

To obtain the fluorescence fingerprint of the "hybrid" duplex, we first measured a series of Cy3-SREseg16-FAM mixtures with an excess of unlabeled complementary strands (cSREseg16) up to a concentration ratio of 1:81. Results are shown in Figure SI-F2. The spectral sets were treated by factor analysis (singular value decomposition), which has demonstrated that they are composed of just two spectral profiles (Figure SI-F3). They apparently correspond to Cy3-SREseg16-FAM hairpins and Cy3-SREseg16-FAM/cSREseg16 duplexes (Cy3-SREseg16-FAM homoduplexes can be for sure neglected). The concentration dependence of V1 and V2 coefficients was fitted according to the formula for equilibrium of heteroduplexes and hairpins (see „Equilibrium equations for fluorescence experiments" section). We used an equilibrium association constant of 470 for Cy3-SREseg16-FAM hairpin, derived from the thermodynamic parameters of SREseg16 determined by means of UV experiment (Table 1). The same value was used for the cSREseg16 hairpin (it was only an estimate, but we realized that variation of this constant within ±50 % did not cause any resolvable changes of resulting spectral isolates). The fit enabled the determination of amounts of hairpins and duplexes (Figure SI-F3, bottom) and isolation of the spectral pattern of the "hybrid" duplex (Figure SI-F4).

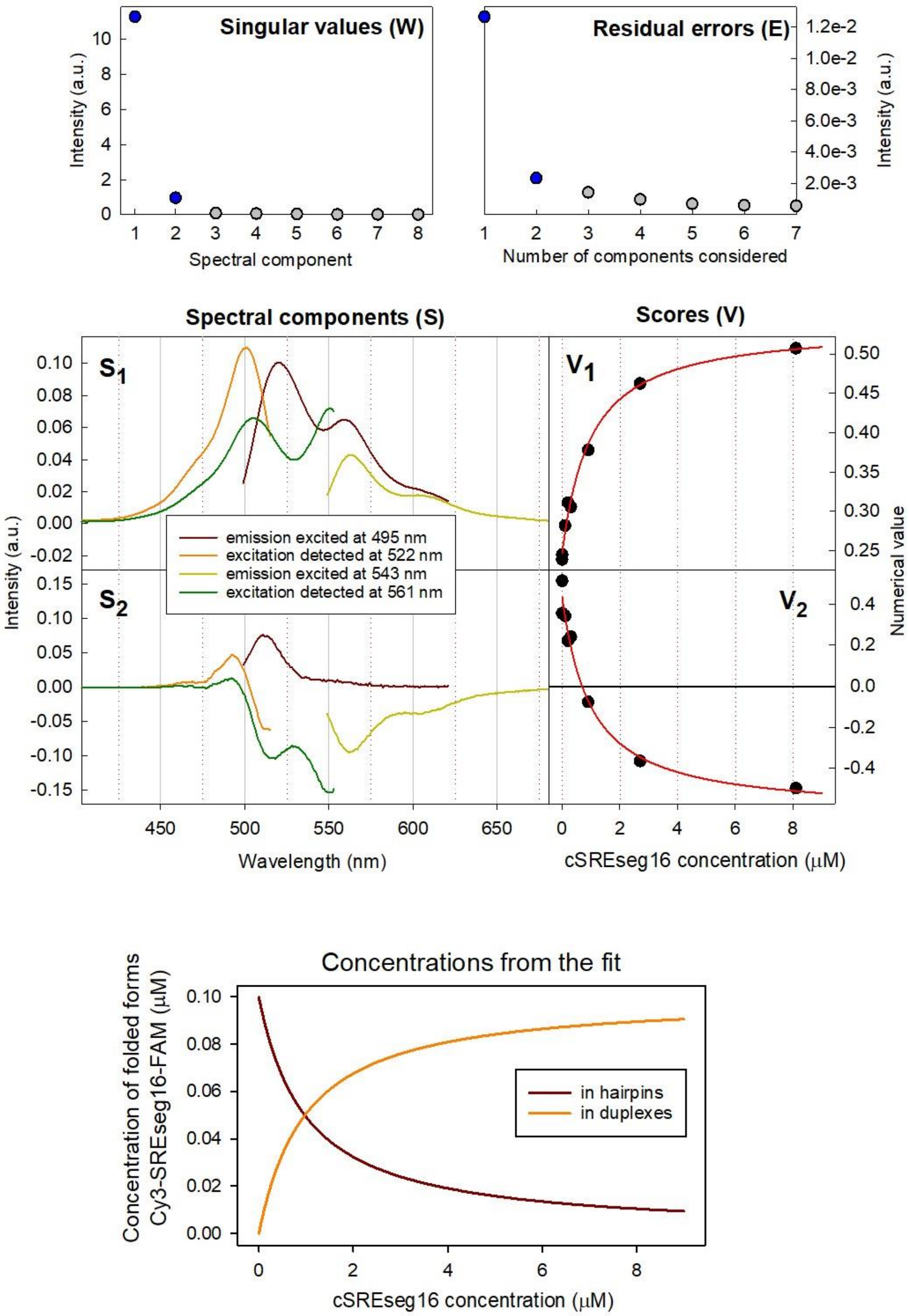

Figure SI-F3. Singular value decomposition of the fluorescence spectra shown in Figure SI-F2. The common fit of V_1 and V_2 coefficients is indicated in red. The bottom graph shows the concentrations of labeled hairpins and "hybrid" duplexes obtained from the fit.

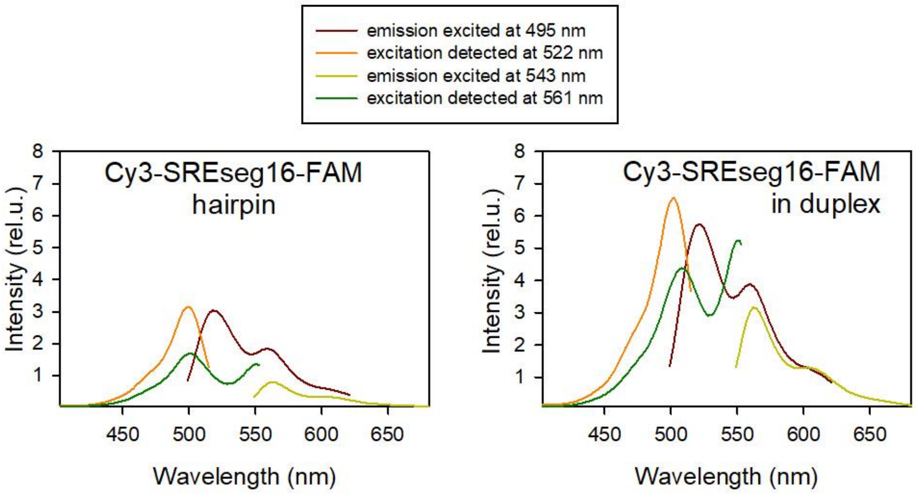

Figure SI-F4. Isolated spectra for Cy3-SREseg16-FAM in hairpin and in "hybrid" duplex with unlabeled complement as a result from SVD.

Then we recorded fluorescence spectra of Cy3-SREseg16-FAM with a very high excess of unlabeled SREseg16 (1:3000 concentration ratio, see Figure SI-F5). The estimated concentration of "hybrid" homoduplexes received by decomposition into the two spectral patterns shown in Fig. SI F4 was only (1.9±1.3) nM. This corresponds to the association constant of homoduplex equal (8±6) μM^{-1}. Then the oligonucleotide amount in homoduplexes should be (at ambient temperature) 6±4 % in the case of highly concentrated samples used in NMR experiments (1 mM).

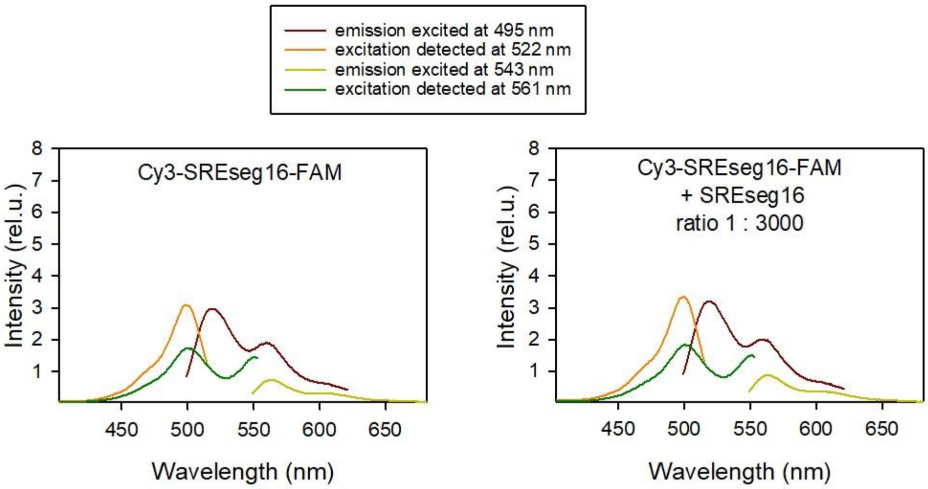

Figure SI-F5. Fluorescence spectra of 0.1µM Cy3-SREseg16-FAM and its mixture with an excess of unlabeled SREseg16.

Equilibrium equations for fluorescence experiments

1. Mixture of two complementary strands that form heteroduplex and individual hairpins

Total concentrations of strands c_1 and c_2, those of hairpins c_{h1} and c_{h2}, of unfolded strands c_{un1} and c_{un2}, and of duplexes c_{du}. The association equilibrium constant of duplexes K_{du}, those of hairpins (estimated as equal) K_{h}. Basic equations:

$$K_{\mathrm{h}} = \frac{c_{\mathrm{h1}}}{c_{\mathrm{un1}}} = \frac{c_{\mathrm{h2}}}{c_{\mathrm{un2}}}, K_{\mathrm{du}} = \frac{c_{\mathrm{du}}}{c_{\mathrm{un1}} . c_{\mathrm{un2}}}$$

$$c_1 = c_{\mathrm{un1}} + c_{\mathrm{h1}} + c_{\mathrm{du}}, \qquad c_2 = c_{\mathrm{un2}} + c_{\mathrm{h2}} + c_{\mathrm{du}}$$

Solution:

$$c_{\mathrm{h1}} = K_{\mathrm{h}} \frac{-(1+K_{\mathrm{h}})^2 + K_{\mathrm{du}}(c_1 - c_2) + \zeta}{2K_{\mathrm{du}}(1+K_{\mathrm{h}})} \qquad c_{\mathrm{h2}} = K_{\mathrm{h}} \frac{-(1+K_{\mathrm{h}})^2 + K_{\mathrm{du}}(c_2 - c_1) + \zeta}{2K_{\mathrm{du}}(1+K_{\mathrm{h}})} \qquad c_{\mathrm{du}} = \frac{(1+K_{\mathrm{h}})^2 + K_{\mathrm{du}}(c_1 + c_2) - \zeta}{2K_{\mathrm{du}}}$$

$$c_{\mathrm{un1}} = \frac{-(1+K_{\mathrm{h}})^2 + K_{\mathrm{du}}(c_1 - c_2) + \zeta}{2K_{\mathrm{du}}(1+K_{\mathrm{h}})} \qquad c_{un2} = \frac{-(1+K_{\mathrm{h}})^2 + K_{\mathrm{du}}(c_2 - c_1) + \zeta}{2K_{\mathrm{du}}(1+K_{\mathrm{h}})} \quad ,$$

where $\zeta = \sqrt{(1+K_{\mathrm{h}})^4 + 2(1+K_{\mathrm{h}})^2 K_{\mathrm{du}}(c_1 + c_2) + {K_{\mathrm{du}}}^2 (c_1 - c_2)^2}$.

<u>2. Mixture of two oligonucleotides of the same sequence, but the first double labeled (A) and the second unlabeled (B).</u>

The solution can contain labeled hairpins (hA), unlabeled hairpins (hB), homoduplexes of two labeled strands (duAA), homoduplexes of two unlabeled strands (duBB), and "hybrid" homoduplexes of one labeled and one unlabeled strand (duAB).

Concentrations c_{A} and c_{B}, association equilibrium constants of hairpins K_{h} and of homoduplexes K_{du}.

Basic equations ($c_{0\mathrm{A}}$ and $c_{0\mathrm{B}}$ are concentrations of unfolded strands):

$$K_{\mathrm{h}} = \frac{c_{\mathrm{hA}}}{c_{\mathrm{unA}}} = \frac{c_{\mathrm{hB}}}{c_{\mathrm{unB}}}, K_{\mathrm{du}} = \frac{c_{\mathrm{duAA}}}{{c_{\mathrm{unA}}}^2} = \frac{c_{\mathrm{duBB}}}{{c_{\mathrm{unB}}}^2} = \frac{c_{\mathrm{duAB}}}{2c_{\mathrm{unA}} c_{unB}}$$

$$c_{\mathrm{A}} = c_{\mathrm{unA}} + c_{\mathrm{hA}} + 2c_{\mathrm{duAA}} + c_{\mathrm{duAB}}, c_{\mathrm{B}} = c_{\mathrm{unB}} + c_{\mathrm{hB}} + 2c_{\mathrm{duBB}} + c_{\mathrm{duAB}}$$

This set of equations leads to an equation, which must be solved numerically to get c_{duAB} or K_{du}:

$$\left[\sqrt{(1+K_{\mathrm{h}})^2 + 8K_{\mathrm{du}}(c_{\mathrm{A}} - c_{\mathrm{duAB}})} - (1+K_{\mathrm{h}})\right]\left[\sqrt{(1+K_{\mathrm{h}})^2 + 8K_{\mathrm{du}}(c_{\mathrm{B}} - c_{\mathrm{duAB}})} - (1+K_{\mathrm{h}})\right] = 8K_{\mathrm{du}} c_{\mathrm{duAB}} \quad .$$

Reference

Dietrich 2002

Dietrich A., Buschmann V., Müller C., and Sauer M. (2002) Fluorescence resonance transfer (FRET) and competing processe in donor-acceptor substituted DNA strands: a comparative study of ensemble and single-molecule data. *Rev. Mol. Biotechnol.* **82**, 211-231.
